# Discoidin domain Receptor 2: A determinant of metabolic syndrome-associated arterial fibrosis in non-human primates

**DOI:** 10.1371/journal.pone.0225911

**Published:** 2019-12-05

**Authors:** Mereena George Ushakumary, Mingyi Wang, Harikrishnan V, Allen Sam Titus, Jing Zhang, Lijuan Liu, Robert Monticone, Yushi Wang, Julie A. Mattison, Rafael de Cabo, Edward G. Lakatta, Shivakumar Kailasam

**Affiliations:** 1 Division of Cellular and Molecular Cardiology, Sree Chitra Tirunal Institute for Medical Sciences and Technology, Trivandrum, Kerala, India; 2 Laboratory of Cardiovascular Science, National Institute on Aging, National Institute on Aging/National Institutes of Health, Baltimore, Maryland, United States of America; 3 Department of Cardiology, The First Hospital of Jilin University, Changchun, China; 4 Translational Gerontology Branch, National Institute on Aging, National Institute on Aging/National Institutes of Health, Baltimore, Maryland, United States of America; Albert Einstein College of Medicine, UNITED STATES

## Abstract

Collagen accumulation and remodeling in the vascular wall is a cardinal feature of vascular fibrosis that exacerbates the complications of hypertension, aging, diabetes and atherosclerosis. With no specific therapy available to date, identification of mechanisms underlying vascular fibrogenesis is an important clinical goal. Here, we tested the hypothesis that Discoidin Domain Receptor 2 (DDR2), a collagen-specific receptor tyrosine kinase, is a determinant of arterial fibrosis. We report a significant increase in collagen type 1 levels along with collagen and ECM remodeling, degradation of elastic laminae, enhanced fat deposition and calcification in the abdominal aorta in a non-human primate model of high-fat, high-sucrose diet (HFS)-induced metabolic syndrome. These changes were associated with a marked increase in DDR2. Resveratrol attenuated collagen type I deposition and remodeling induced by the HFS diet, with a concomintant reduction in DDR2. Further, in isolated rat vascular adventitial fibroblasts and VSMCs, hyperglycemia increased DDR2 and collagen type I expression via TGF-β1/SMAD2/3, which was attenuated by resveratrol. Notably, gene knockdown and overexpression approaches demonstrated an obligate role for DDR2 in hyperglycemia-induced increase in collagen type I expression in these cells. Together, our observations point to DDR2 as a hitherto unrecognized molecular link between metabolic syndrome and arterial fibrosis, and hence a therapeutic target.

## Introduction

Vascular fibrosis is characterized by accumulation and remodeling of the extracellular matrix (ECM) within the vascular wall, which leads to intimal-medial thickening, reduced lumen diameter and compromised vascular resilience[1]. Fibrosis-related changes in the arterial wall lead to arterial stiffness and exacerbate the complications of chronic metabolic stress[2], advancing age[3] and conditions such as atherosclerosis[4], hypertension[5] and diabetes mellitus[6]. Understanding the pathogenesis of vascular fibrosis is clearly a major clinical goal in a setting of alarming global prevalence of cardiovascular diseases and associated morbidity and mortality.

Adventitial fibroblasts and vascular smooth muscle cells (VSMCs) act as principal effectors in the progression of vascular fibrosis. They are the predominant source of type I and III collagens within the vascular media and adventitia and moderate collagen homeostasis. In the steady state, collagen and elastin help maintain normal vascular structure and function by providing tensile strength and elasticity[7]. In response to hemodynamic alterations[8] and cardiovascular risk factors such as hypertension[9], hyperglycemia[10] and dyslipidemia[11] associated with metabolic syndrome, adventitial fibroblasts and VSMCs initially promote adaptive structural modifications involving alterations in ECM turnover to meet altered functional demands. However, prolonged exposure to pathological, physical and chemical factors causes excessive deposition of ECM proteins, particularly collagen, by adventitial fibroblasts in the vascular adventitia and VSMCs in the vascular medial layer, resulting in vascular fibrosis that offsets the benefits of the initial adaptive remodeling process. As a major contributor to the initiation and progression of several vascular diseases[12–14], vascular fibrosis leads eventually to impairment of target organs such as the heart, brain and kidneys. Identification of cellular contributors to vascular fibrosis could potentially aid in the development of novel therapeutic strategies for the treatment of life-threatening vascular conditions.

In this regard, the role of collagen-collagen receptor interaction as a key determinant of collagen production and matrix remodeling is an emerging paradigm. Discoidin Domain Receptor 2 (DDR2), a collagen receptor tyrosine kinase expressed exclusively in cells of mesenchymal origin such as fibroblasts and VSMCs[15], is reported to significantly impact tissue response to injury [16]. Further, it has been implicated in fundamental cellular processes such as cell survival[17], proliferation, migration[18] and differentiation[19,20]. These observations provide compelling rationale for the hypothesis that DDR2 could be a critical determinant of metabolic syndrome-associated vascular fibrosis.

Our previous studies on a clinically relevant adult male rhesus monkey model of high fat, high sucrose (HFS)-induced metabolic syndrome demonstrated an increase in body weight and cholesterol, loss of endothelial cell integrity, lipid and macrophage infiltration, and calcification of the arterial wall, driven by genomic and proteomic signatures of oxidative stress and inflammation[21]. Notably, the HFS diet induced central arterial wall stiffening and elevated pulse wave velocity (PWV). A major contributor to arterial stiffness, which is an important determinant of elevated central systolic blood pressure and cardiovascular outcomes, is vascular fibrosis and ECM remodeling. Based on investigations on the same non-human primate model of diet induced metabolic syndrome in tandem with vascular cells exposed to hyperglycemic conditions in vitro, the present study proposes that DDR2 may be a molecular link between hyperglycemia and arterial fibrosis and a potential drug target in the control of adverse arterial remodeling associated with metabolic stress and, possibly, other vascular pathologies. The findings also show that resveratrol, a polyphenol, may attenuate adverse collagen remodeling in the vascular wall via its inhibitory action on DDR2 expression.

## Materials and methods

### Materials

PD169316 and SIS3 were from Calbiochem (San Diego, USA). Collagenase type II and elastase (lyophilized) were from Worthington (Columbus, USA). Glucose, SB431542 hydrate, Resveratrol, Transforming Growth Factor-β1 (TGF-β1, Catalogue number T7039) and Medium199 (M199) were from Sigma-Aldrich (St. Louis, USA). Primary antibodies against DDR2 (catalog number 12133S; rabbit monoclonal; 1:1000), TGF-β1 (catalog number 3711; rabbit polyclonal; 1:1000), phospho-ERK1/2 MAPK (catalog number 4370; rabbit monoclonal; 1:1000) and total ERK1/2 MAPK (catalog number 4370; rabbit monoclonal; 1:1000) were obtained from Cell Signaling Technology (Danvers, USA). COL1A1 (catalog number sc-8784; rabbit polyclonal; 1:1000) was from Santa Cruz Biotechnology (Dallas, USA). The following antibodies were used for detection of collagen type I, type III and DDR2 protein in monkey aortic tissue homogenates—Collagen I Polyclonal Antibody (1:500; Catalog #: PA1-35998; Invitrogen Thermo Fisher Scientific, US). Collagen III Polyclonal Antibody (1:1000; Catalog #: PA1-28870; Invitrogen Thermo Fisher Scientific, US). Anti-DDR2 Antibody (1:800; Catalog #: MABT322 from: Sigma-Aldrich, US). Anti-rabbit IgG peroxidase secondary antibody (A0545; goat polyclonal; 1:10,000) and Anti-mouse IgG peroxidase secondary antibody (A9044; rabbit polyclonal; 1:10,000) were from Sigma-Aldrich (St. Louis, USA). Random primers, Reverse transcriptase, RNAase inhibitor and dNTPs were obtained from Promega Corporation (Madison, USA). DDR2 siRNA, Col1a1 siRNA and TGF-β1 siRNA were from Ambion(Waltham, USA). Lipofectamine was from Invitrogen (Waltham, USA). Opti-MEM and fetal bovine serum were from GIBCO (Waltham, USA). Rat DDR2/CD167b Gene ORF cDNA clone expression plasmid and FLAG-tagged Rat DDR2 cDNA clone expression plasmid were obtained from Sino Biologicals (Beijing, China). All cell culture ware was purchased from BD Falcon (Corning, USA).

### Ethics statement

Handling of Sprague Dawley rats and experimental procedures were in accordance with the US National Institutes of Health guidelines for animal care and use (No. 85–23, revised 2011) with Institutional Animal Ethics Committee approval (SCT/IAEC-023/June/2012/77). Animals were euthanized (Thiopentone sodium, 5–10 mg/kg, i.p). The aorta was excised and used for isolation of adventitial fibroblasts and VSMCs. Experiments on non-human primates were approved by the Animal Care and Use Committee of the NIA Intramural Research Program. These experiments were performed in accordance with the guidelines and regulations of Animal Care and Use Committee of the NIA Intramural Research Program.

### General welfare of the non-human primates

Monkeys were housed individually in standard nonhuman primate caging on a 12h light/12h dark cycle, room temperature (78±2°F), and humidity at 60±20% at the NIH Animal Center, Poolesville, MD. All animals were considered healthy with no cardiovascular abnormalities. The animal center is fully accredited by the American Association for Accreditation of Laboratory Animal Care. One pairing was maintained throughout the study; all other monkeys had extensive visual, auditory, and olfactory but limited tactile contact with monkeys housed in the same room. Monkeys received 2 meals per day at estimated *ad libitum* levels throughout the study. Water was always available *ad libitum*. Monkeys were monitored minimally 3 times daily by trained animal care staff. Anesthesia was induced with Ketamine (7–10 mg/kg, IM) and, following blood collection, the monkeys were deeply anesthetized with a lethal dose of sodium pentobarbital (50 mg/kg, IP). Once maximally sedated, the monkeys were perfused with cold lactated Ringer’s solution until death. Tissues were harvested immediately.

### Animals and diet

During baseline assessments, all monkeys were maintained on a commercially available closed formula monkey chow (TestDiet^®^ #5038 Purina Mills, Richmond, IN). After baseline assessment, 14 male rhesus monkeys (7–13 years) were quasi-randomized into 2 groups: a HFS diet (n = 10); and the healthy standard diet (n = 4). The SD was a purified biscuit with 13% kcal from fat and less than 5% sucrose by weight. The HFS diet was a specially formulated purified ingredient diet with 42% kcal from fat and approximately 27% sucrose by weight (Harlan, Teklad, Madison, WI). The monkeys were gradually switched to the HFS diet over a 3-week period. All groups received 2 meals per day of the specified diet for 2 years in allotments that represented *ad libitum* feeding, ensuring iso-caloric consumption across groups. The study also included a resveratrol group (n = 10), provided with a daily dietary supplementation of resveratrol (80 mg during the 1st year and 480 mg during the 2nd year) along with the HFS diet. Animal experiments were performed under conditions described earlier[21].

### Isolation of rat vascular adventitial fibroblasts and VSMCs

Vascular adventitial fibroblasts and VSMCs were isolated from young adult male Sprague-Dawley rats following protocols previously standardized by us [22]. Aorta was removed from left subclavian origin, washed in HBSS solution and then transferred in a sterile hood into clean 100 mm dish with fresh HBSS. Fat was removed gently under a microscope and incubated in a 35 mm dish containing 2 ml Digestion Medium I for 30 min at 37°C. Tissue was then placed in fresh HBSS, cut open, separated into the outermost adventitial layer and middle VSMCs after scrapping out the endothelium, and incubated in Digestion Medium II for 90 min at 37°C. After incubation, an equal volume of M199 containing 10% FBS was added to the dish and mixed well for proper dissociation. Cells were collected by filtering through a cell strainer (70 μm) and centrifuged at 1500 rpm for 5 min. The cell pellet was collected and re-suspended in 0.5 ml of M199 containing 10% FBS and seeded on 12-well plate. At 24 h after isolation, the supernatant containing unattached cells was discarded and the cells were incubated with M199 containing 10% FBS in a CO_2_ incubator at 37°C. Cultures of adventitial fibroblasts and VSMCs were characterized and checked for cross-contamination by immunocytochemistry. Adventitial fibroblasts stained positive for DDR2 and negative for Desmin (S1 Fig). Calponin was used to characterize and ascertain the purity of VSMC cultures (S2 Fig).

Cells from passage 3–4 were used for the experiments. Hyperglycemic conditions were generated by exposing the cells to 25 mM glucose, with 25 mM mannitol as osmolarity control.

### Real-time polymerase chain reaction analysis

Sub-confluent cultures of adventitial fibroblasts and VSMCs were subjected to the indicated treatments and total RNA was isolated using TRI reagent, according to the manufacturer's instructions. Following DNase I treatment, 2 μg of total RNA was reverse transcribed to cDNA with random primers and M-MLV reverse transcriptase. TaqMan quantitative RT-PCR analysis was carried out using the ABI prism 7500 Sequence Detection System (Applied Biosystems, CA) with specific FAM-labeled probes. PCR reactions were performed over 40 cycles, as per the manufacturer's instructions. DDR2 and collagen mRNA expression levels were normalized to β-actin and 18s rRNA, respectively.

### Western blot analysis

Sub-confluent cultures of adventitial fibroblasts and VSMCs in serum-free M199 were treated with high glucose, and relative collagen and DDR2 protein expression was determined by western blot analysis following standard protocols, using β-actin as loading control. Enhanced chemiluminescence reagent was used to detect the proteins, and protein expression was quantified by densitometric scanning (Bio-Rad Laboratories). The following protocol was employed for western blot analysis on monkey aortic tissue homogenate. Immediately after harvesting, monkey abdominal aortic tissue was rinsed in 1X PBS, frozen in liquid nitrogen and pulverized in the presence of T-PER Tissue Protein Extraction Reagent. Lysates were centrifuged for 10 min to remove debris and 40 μg of total homogenous arterial protein was resolved by SDS-PAGE and transferred onto PVDF membrane. The transferred membranes were incubated in 3% non-fat milk containing primary antibodies at 4°C overnight. HRP-conjugated IgG were used as secondary antibodies and detected with Super Signal West Pico Chemiluminescent Substrate. Densitometric analysis was performed on the protein bands from the aortae of 3 monkeys from each group. Beta-actin immunoblotting was used as a protein loading control.

### RNA interference

RNA interference protocol was as reported by us earlier. Briefly, cells were seeded on 12-well plates at 8×10^4^ cells/well. After 24 h, the cells were incubated in Opti-MEM with Ambion pre-designed Silencer Select siRNA [5 pmol DDR2, 5 pmol collagen α1 type1, 5 pmol TGF-β1 or scrambled siRNA, control] and Lipofectamine 2000 (2 μl) for 19 h. Following an additional incubation in M199 with 10% FBS for 12 h, the cells were treated with high glucose for the indicated duration. Cell lysate was prepared in SDS lysis buffer, denatured and used for western blot analysis.

### Over-expression of DDR2

Strong constitutive expression of DDR2 was achieved under a pCMV promoter, purchased as cDNA clone from Sinobiologicals, China (Rat DDR2/CD167b Gene ORF cDNA). The DDR2 clone size was verified by PCR amplification of the primers provided in the kit. The size of the plasmid was further analyzed by single site restriction digestion at the Mlu-1 site. DDR2 over-expression plasmid cocktail was prepared with 1 μg plasmid and 3 μl Lipofectamine 2000 in 100 μl Opti-MEM, which was added to the cultures for incubation in M199+10% FBS for 8 hours. After a recovery period of 12 hours, cells were pre-treated with the TGF-β1 inhibitor (SB431542 hydrate) or resveratrol followed by HG treatment. Cultures of adventitial fibroblasts and VSMCs were transfected with FLAG-tagged DDR2 plasmid vector. The number of cells staining positive for FLAG in 4–5 fields was analyzed by immunocytochemistry (S3A and S3B Fig) to ascertain overexpression efficiency, which was found to be >80%.

### Histochemistry, immunohistology and semi-quantification analysis

In 4–6 consecutive 5 μm paraffin embedded aortic cross-sections of monkey abdominal aortae, we performed Elastica van Gieson (EVG) (to determine elastin degradation), picrosirius red (to determine fibrosis), collagen (to determine collagen deposition), collagen type I and DDR2 antibodies stain (to determine collagen type I deposition and DDR2 expression, respectively), and Masson Trichrome stain (to determine extracellular matrix remodeling). In 4–6 consecutive 10 μm frozen aortic cross-sections, we performed Oil Red O (to determine fat deposits) and Alizarin Red staining (to determine calcium deposits), according to the modified protocols as reported in prior studies[21]. 4 fields each from 4 abdominal aortic cross-sections from each animal were analyzed. Methods and criteria of semi-quantification and quantification of histochemical and immunobiological staining are shown in Table 1.

**Table 1:** Semi-quantification and quantification criteria for immunohistochemistry (Figs 7 and 8).

Parameter	Score/Grade
Fibrosis	% area of ECM determined by Masson Staining and % area of collagen determined by picrosirius red staining
Collagen type I deposition	% area of collagen type I staining
DDR2-positive cells	Score 1: no/mild DDR2 staining in vascular cells Score 2: <10% DDR2 staining in vascular cells Score 3: 10 to 50% DDR2 staining in vascular cells Score 4: > 50% DDR2 staining in vascular cells.
Calcification	Score 0: Absence or mild presence in vascular walls Score 1: focal present in the media in vascular walls Score 2: Multifocal presence in the media in vascular wall Score 3: Multifocal presence in both the intima and media in vascular walls Score 4: Diffuse presence in both intima and media in vascular walls
Fat deposition	Score 0: Absence or mild presence in vascular walls Score 1: focal moderate present in the intima in vascular walls Score 2: Multifocal moderate to severe presence in the intima in vascular wall Score 3: Diffuse presence in both intima and media in vascular walls.
Elastin fragmentation	Score 1: Absence of facture in vascular walls Score 2: Focal fracture in vascular walls Score 3: Multifocal fracture in vascular wall Score 4: Diffuse fracture in vascular walls

### Statistical analysis

Data were expressed as Mean±S.D. Statistical analysis was performed using Student's *t* test. p≤0.05 was considered significant. Data were also analyzed by one-way ANOVA and p≤0.05 was considered statistically significant. The data presented are biological replicates that are representative of three independent experiments where n = 3. In vivo, experiments were statistically analyzed using One-way ANOVA and Kruskal-Wallis with Post-hoc Dunn’s test and p≤0.05 was considered statistically significant. For in vivo experiments, data were expressed as Mean±S.E.M [SD (n = 4),HFS (n = 10), and HFS+RESV (n = 10)].

## Results

### DDR2 mediates TGF-β1-dependent increase in collagen type I gene expression in advential fibroblasts exposed to high glucose

**Fig 1:**
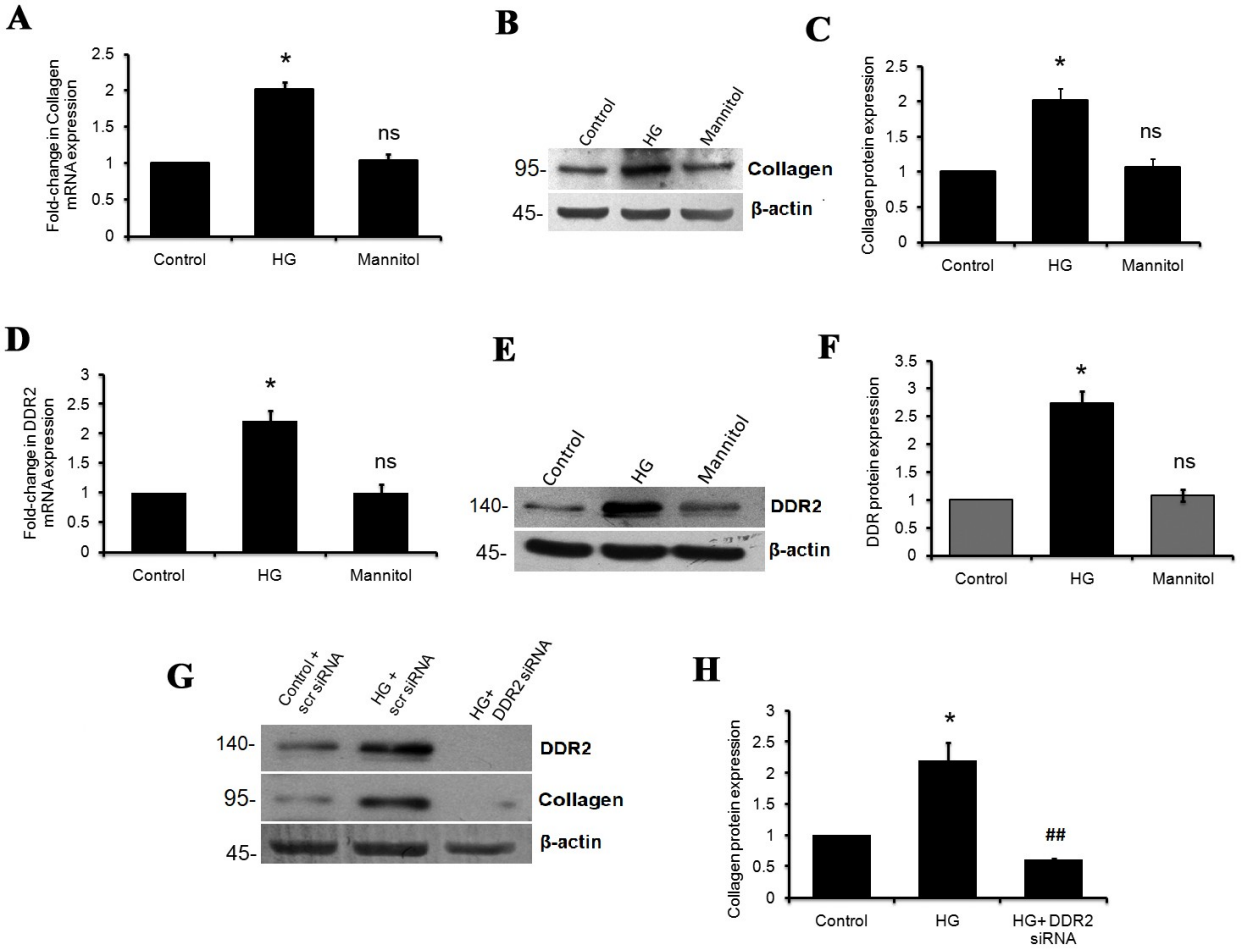
HG enhances collagen type I and DDR2 gene expression in vascular adventitial fibroblasts. Sub-confluent quiescent cultures of vascular adventitial fibroblasts in M199 were stimulated with HG (25mM). **(A)** Collagen type I alpha 1 mRNA levels were determined by Taqman Real-time PCR analysis at 6 h of HG treatment. 18S rRNA served as the endogenous control. *p<0.05 vs. control, ns- not significant vs. control (One-way ANOVA p< 0.05). **(B and C)** Protein was isolated and subjected to western blot analysis for detection of collagen type I alpha 1, with β-actin as loading control. *p< 0.05 vs. control, ns- not significant vs. control (One-way ANOVA p<0.05). **(D-F)** Sub-confluent quiescent cultures of vascular adventitial fibroblasts in M199 were stimulated with HG (25mM). **(D)** DDR2 mRNA levels were determined by Taqman quantitative real-time PCR analysis at 12 h of HG treatment, with β-actin as loading control. *p< 0.05 vs. control, ns- not significant vs. control (One-way ANOVA p< 0.05). (**E and F)** Protein was isolated and subjected to western blot analysis for detection of DDR2, with β-actin as loading control. *p< 0.05 vs. control, ns- not significant vs. control (One–way ANOVA p< 0.05). (**G and H)** Regulatory relationship between DDR2 and collagen type I in HG-treated adventitial fibroblasts. Adventitial fibroblasts were transiently transfected with DDR2 siRNA or scrambled siRNA. Following exposure of the transfected cells to HG for 12 h, collagen type I alpha 1 protein expression was examined by western blot analysis and normalized to β-actin. *p< 0.05 vs. control, ## p< 0.01 vs. HG (One-way ANOVA p< 0.05). Data are representative of three independent experiments, n = 3. Error bars represent SD.

The effect of 25 mM glucose (HG) on collagen type I alpha1 mRNA and protein expression in vascular adventitial fibroblasts was determined by real-time PCR and western blot analyses, respectively. A 2-fold increase in collagen type I alpha 1 mRNA (Fig 1A) and protein expression (Fig 1B and 1C) was observed in cells exposed to hyperglycemic conditions for 12 h.

HG also caused a 2-fold increase in DDR2 mRNA expression (Fig 1D) and a 2.5-fold increase in DDR2 protein expression (Fig 1E and 1F) in these cells. The effect of HG on collagen type 1 alpha 1 and DDR2 expression was sustained at 48 h (S4A and S4B Fig).

To test the possibility that a regulatory relationship exists between DDR2 and collagen type 1 in HG-stimulated adventitial fibroblasts, cells were transfected with DDR2 siRNA as described under Methods. After confirming knockdown, collagen type I alpha1 expression was determined following treatment with HG. RNA interference-based inhibition of DDR2 attenuated the stimulatory effect of HG on collagen type I alpha 1 expression (Fig 1G and 1H), indicating a regulatory role for DDR2 in collagen type I gene expression in HG-treated adventitial fibroblasts. The regulatory role of DDR2 in collagen was also observed under basal conditions (S4E Fig).

**Fig 2:**
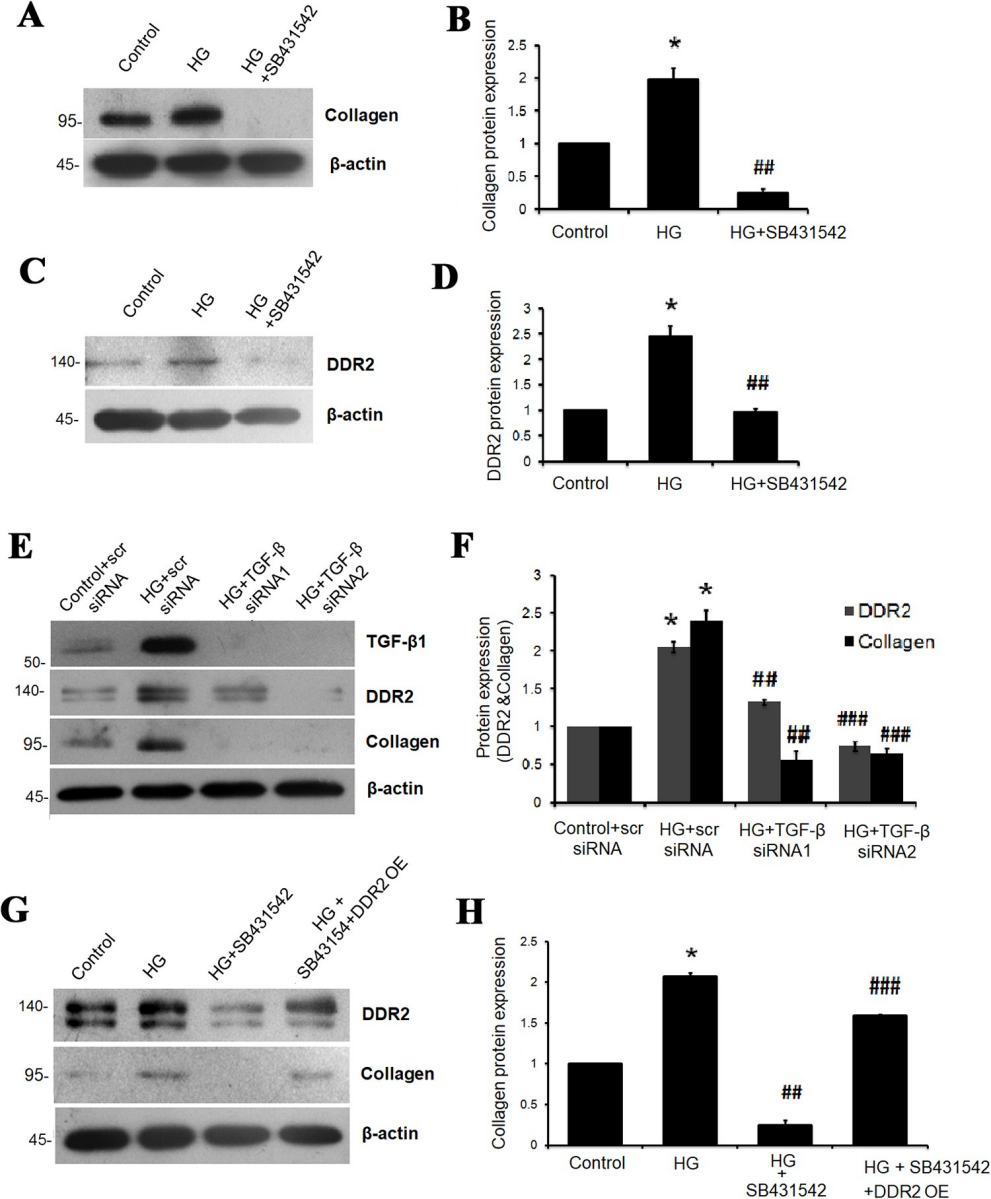
TGF-β1 mediates HG-induced DDR2 and collagen gene expression. **(A-D)** Inhibition of TGF-β1 abolished HG-induced collagen type I alpha 1 and DDR2 gene expression. Following pre-treatment of cells with TGF-β1 inhibitor, SB431542, for 1 h and subsequently with HG for 12 h, **(A and B)** collagen type I alpha 1 protein expression *p< 0.05 vs. control, ##p< 0.01 vs. HG (One-way ANOVA p<0.05), and **(C and D)** DDR2 protein expression *p< 0.05 vs. control, ##p< 0.01 vs. HG (One-way ANOVA p<0.05) were examined by western blot analysis, with β-actin as loading control. **(E and F)** RNAi-mediated silencing of TGF-β1 confirmed its role in regulating DDR2 and collagen gene expression. Vascular adventitial fibroblasts were transiently transfected with TGF-β1 siRNA 1 and 2 (5 pmol) or scrambled siRNA prior to treatment with HG for 12 h. DDR2 (*p< 0.05 vs. control, ## p< 0.01 vs. HG, ### P<0.001 vs. HG) and collagen type I alpha 1 (*p< 0.05 vs. control, ##p< 0.01 vs. HG, ###p< 0.001 vs. HG) protein expression was examined, with β-actin as loading control (One-way ANOVA p<0.05). (**G and H)** Effect of DDR2 over-expression on HG-induced collagen type I alpha 1 gene expression in TGF-β1-inhibited adventitial fibroblasts. The ready-to-transfect DDR2 plasmid cocktail was added to the cultures for incubation in M199 for 8 h. After a recovery period of 12 h, cells were pre-treated with SB431542 followed by HG treatment. First lane confirms over-expression of DDR2. *p< 0.05 vs. control, ##p< 0.01 vs. HG, ###p< 0.001 vs. HG+SB431542 (One-way ANOVA p< 0.05). β-actin was used as the loading control. Data are representative of three independent experiments, n = 3. Error bars represent SD.

Inhibition of TGF-β1 using SB431542 abolished HG-induced increase in collagen type I alpha 1 and DDR2 expression (Fig 2A–2D), which was further confirmed by RNA interference-based TGF-β1 knockdown (Fig 2E and 2F). Knockdown of TGF-β1 also attenuated basal expression levels of collagen type I alpha 1 and DDR2 (S4G Fig). Importantly, however, the decrease in collagen type I alpha 1 expression upon TGF-β1 inhibition was not observed in cells over-expressing DDR2 (Fig 2G and 2H). Together, these data show that TGF-β1 acts via DDR2 to enhance collagen type I expression in adventitial fibroblasts exposed to HG.

**Fig 3:**
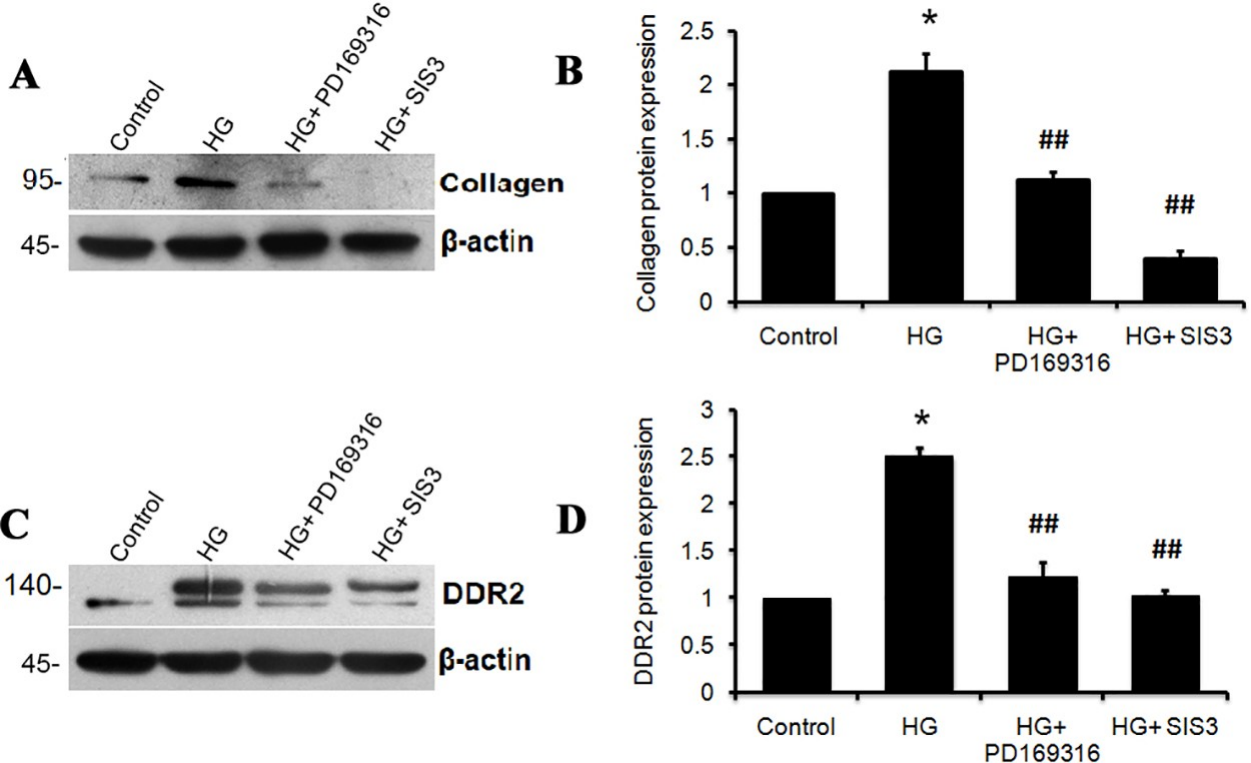
SMAD2/3 inhibition attenuates HG-induced DDR2 and collagen expression. Sub-confluent quiescent cultures of vascular adventitial fibroblasts in M199 were pre-treated with SMAD2 inhibitor, PD169316, and SMAD3 inhibitor, SIS3, for 1 h and subsequently with HG for 12 h. Post-HG treatment, protein was isolated and subjected to western blot analysis for detection of: (**A and B)** Collagen type 1 alpha 1; *p< 0.05 vs. control, ##p< 0.01 vs. HG (One-way ANOVA p< 0.05) and (**C and D)** DDR2; *p< 0.05 vs. control, ##p< 0.01 vs. HG. (One-way ANOVA p< 0.05). β-actin was used as loading control. Data are representative of three independent experiments, n = 3. Error bars represent SD.

**Fig 4:**
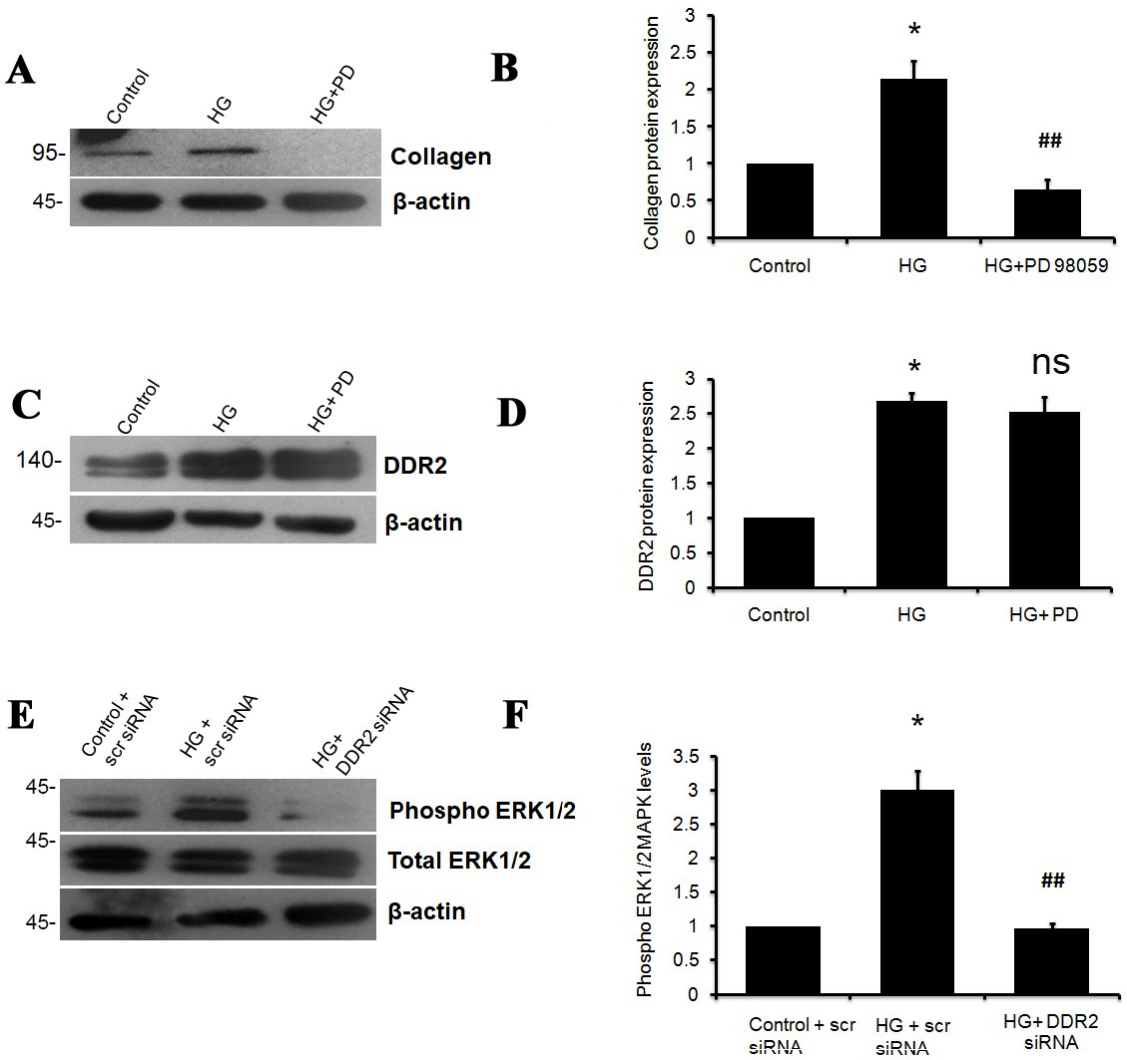
ERK1/2 MAPK mediates DDR2-dependent collagen gene expression in HG-treated cells. **(A-D)** Sub-confluent quiescent cultures of vascular adventitial fibroblasts in M199 were pre-treated with ERK1/2 inhibitor, PD98050, for 1 h and subsequently with HG for 12 h. Protein was isolated at 12 h post-HG treatment and subjected to western blot analysis for detection of: (**A and B)** Collagen type I alpha 1 protein expression; *p< 0.05 vs. control, ##p< 0.01 vs. HG (One-way ANOVA p< 0.05), and (**C and D)** DDR2 protein expression; *p<0.05 vs. control, ns- not significant vs. HG (One-way ANOVA p< 0.05). β-actin was used as the loading control. (**E and F)** DDR2 knockdown abolished the HG-induced activation of ERK1/2, determined by western blot analysis using anti-ERK1/2 antibody. Phospho ERK1/2 levels were normalized to total ERK1/2 levels. *p< 0.05 vs. control, ##p< 0.01 vs. HG (One-way ANOVA p< 0.05). Data are representative of three independent experiments, n = 3. Error bars represent SD.

Inhibition of SMAD 2 and 3 using PD169316 and SIS3, respectively, was found to attenuate HG-induced collagen type I alpha 1 (Fig 3A and 3B) and DDR2 (Fig 3C and 3D) expression in adventitial fibroblasts, indicating the regulatory role of TGF-β1/SMAD2/3 signaling in HG-induced increase in DDR2 and collagen expression. ERK1/2 inhibition using PD98059 attenuated HG-induced expression of collagen type 1 alpha 1 (Fig 4A and 4B) but not DDR2 (Fig 4C and 4D). Further, DDR2 knockdown prevented HG-induced ERK1/2 phosphorylation (Fig 4E and 4F), indicating that DDR2-dependent ERK1/2 activation enhances collagen type I expression in cells exposed to HG.

### Resveratrol prevents hyperglycemia-induced DDR2 and collagen type I in vascular adventitial fibroblasts

**Fig 5:**
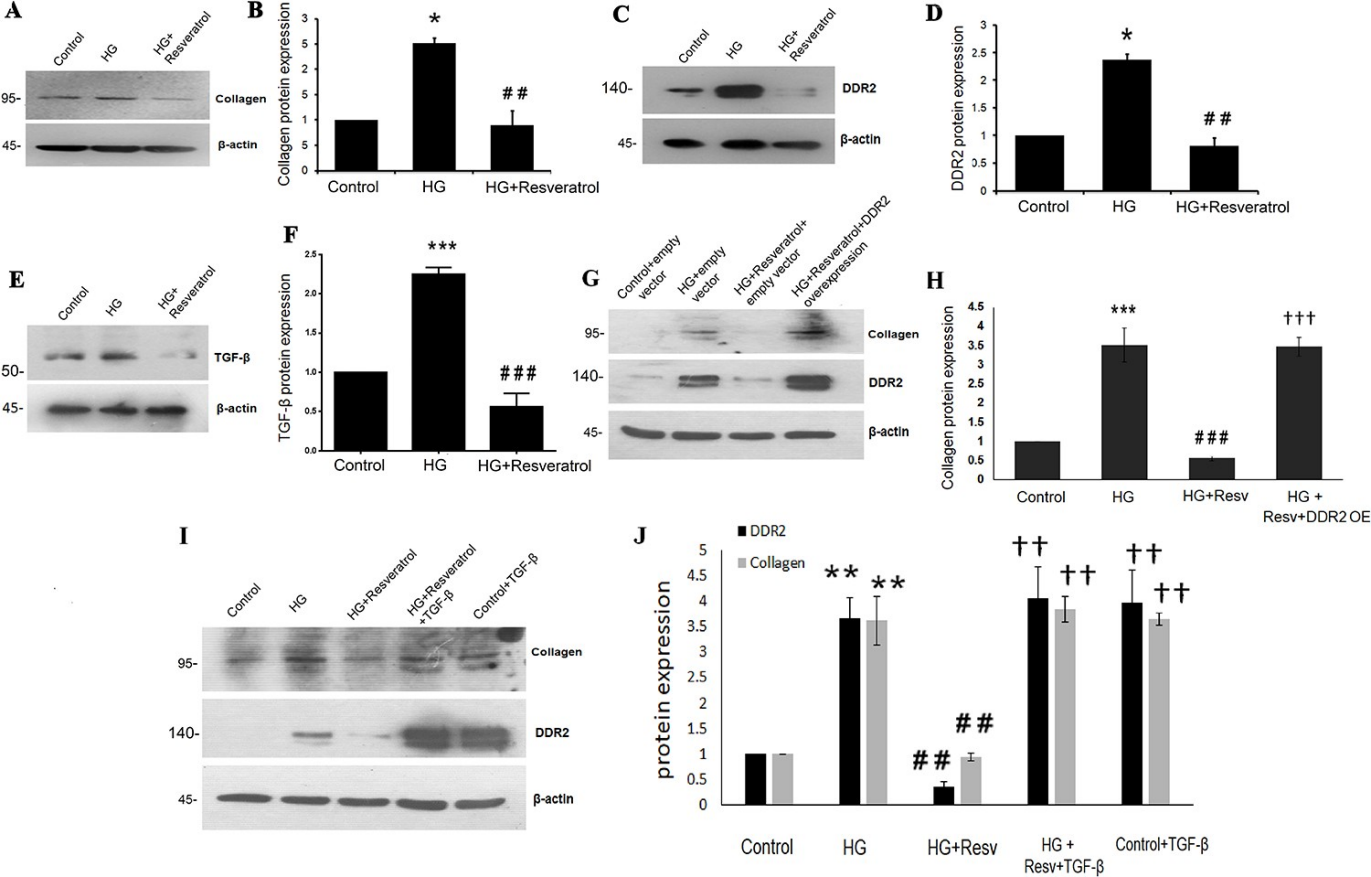
Resveratrol prevents HG-induced collagen type I and DDR2 in vascular adventitial fibroblasts. **(A-F)** Sub-confluent quiescent cultures of vascular adventitial fibroblasts in M199 were pre-treated with 50 μM resveratrol for 1 h and subsequently with HG for 12 h. Protein was isolated at 12 h post-HG treatment and subjected to western blot analysis for detection of: (**A and B)** Collagen type I alpha 1; *p< 0.05 vs. control, ## p< 0.01 vs. HG (One-way ANOVA p< 0.05), and (**C and D)** DDR2; *p< 0.05 vs. control, ## p< 0.01 vs. HG (One-way ANOVA p< 0.05). **(E and F)** TGF-β1; ***p< 0.001 vs. control, ### p< 0.001 vs. HG (One-way ANOVA p< 0.05). β-actin was used as loading control. **(G and H)** Vascular adventitial fibroblasts were transiently transfected with DDR2 overexpression vector. Post-transfection, following revival and serum deprivation, the cells were pre-treated with resveratrol for 1 h, followed by stimulation with HG for 12 h. Collagen type I alpha 1 protein was examined by western blot analysis. ***p< 0.001 vs. control, ### p<0.001 vs. HG, ††† p< 0.001 vs. HG+RESV. **(I and J)** Vascular adventitial fibroblasts were pre-treated with resveratrol for 1 h, followed by stimulation with HG and exogenously added recombinant TGF-β1 (10ng/mL). Protein expression levels of collagen type I alpha 1 and DDR2 were examined at 12 h by western blot analysis. ** p<0.01 vs. control, ## p<0.01 vs. HG, †† p<0.01 vs. HG+RESV. Data are representative of three independent experiments, n = 3. Error bars represent SD.

Pre-treatment of cells with 50 μmol/L resveratrol significantly reduced HG-induced increase in collagen type I alpha 1 expression (Fig 5A and 5B). Resveratrol was also found to prevent HG effect on DDR2 expression (Fig 5C and 5D). It attenuated basal expression levels of collagen type I alpha 1 and DDR2 in vascular adventitial fibroblasts (S4H and S4I Fig). Notably, resveratrol inhibited HG-induced (Fig 5E and 5F) and basal (S4J Fig) TGF-β1 expression in these cells. Interestingly, however, the inhibitory effect of resveratrol on HG-stimulated collagen type I expression was not observed in DDR2-overexpressing cells, showing that attenuation of HG-stimulated collagen type I expression by resveratrol is due to its negative regulation of DDR2 (Fig 5G and 5H). Moreover, exogenous addition of recombinant TGF-β1 (10ng/mL) restored DDR2 and collagen type I alpha 1 expression in resveratrol-treated cells, suggesting that resveratrol inhibits TGF-β1 to attenuate DDR2 and collagen type I expression in response to HG (Fig 5I and 5J).

#### Hyperglycemia stimulates collagen type I and DDR2 expression in VSMCs

**Fig 6:**
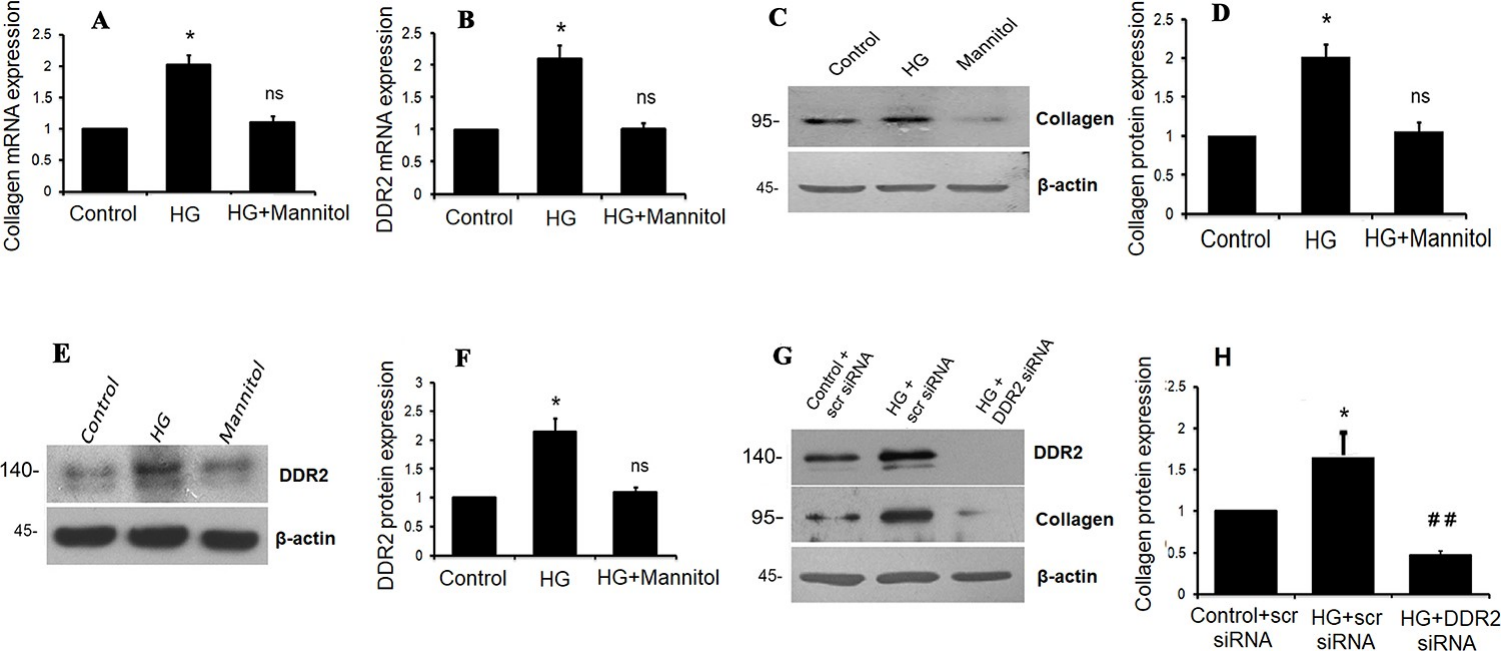
HG stimulates collagen type I and DDR2 in VSMCs. **(A-F)** Sub-confluent cultures of VSMCs in M199 were stimulated with HG (25mM). **(A)** Collagen type I alpha 1 mRNA levels were determined by Taqman real-time PCR at 12 h post-HG treatment, with 18S rRNA as loading control. *p<0.05 vs. control, ns- not significant vs. control (One-way ANOVA p< 0.05). **(B)** DDR2 mRNA levels were determined at 12 h post-HG treatment. *p< 0.05 vs. control, ns- not significant vs. control (One-way ANOVA p< 0.05). **(C and D)** Collagen type I alpha 1 expression was determined by western blot analysis, with β-actin as loading control. *p<0.05 vs. control, ns- not significant vs. control (One-way ANOVA p< 0.05). **(E and F)** DDR2 protein expression was determined by western blot analysis. *p< 0.05 vs. control, ns- not significant vs. control (One-way ANOVA p< 0.05). **(G and H)** Regulatory relationship between DDR2 and collagen type I in HG-treated VSMCs. VSMCs were transiently transfected with DDR2 siRNA or scrambled siRNA. Following exposure of the transfected cells to HG for 12 h, collagen type I alpha 1 protein expression was examined by western blot analysis. DDR2 knockdown using siRNA was validated. *p< 0.05 vs control, ## p< 0.01 vs HG. (One-way ANOVA p< 0.05).

**Fig 7:**
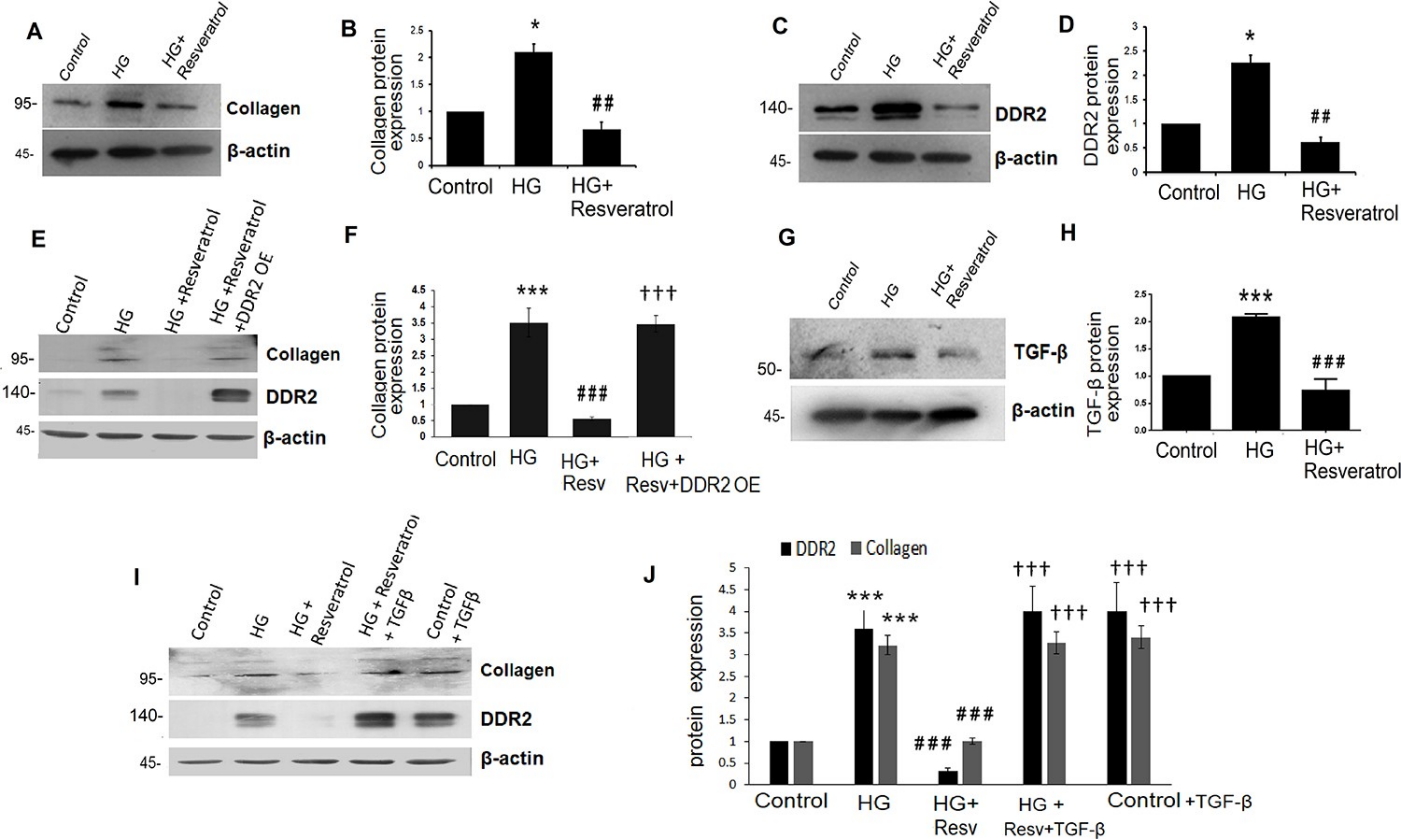
Resveratrol prevents HG-induced collagen type I and DDR2 in VSMCs. (**A-D & G,H)** Resveratrol prevents HG-induced collagen type I alpha 1, DDR2 and TGF-β1 expression in VSMCs. Pre-treatment of cells with 50 μM resveratrol followed by HG for 12 h reduced: **(A and B)** HG-induced increase in collagen type I alpha 1 expression. *p< 0.05 vs control, ## p< 0.01 vs HG (One-way ANOVA p< 0.05). (**C and D)** HG-induced increase in DDR2 expression. *p< 0.05 vs control, ## p< 0.01 vs HG (One-way ANOVA p< 0.05). **(E and F)** Vascular smooth muscle cells were transiently transfected with DDR2 overexpression vector. Post-transfection, following revival and serum deprivation, the cells were pre-treated with resveratrol for 1 h, followed by stimulation with HG for 12 h. Collagen type I alpha 1 protein was examined by western blot analysis.*** p<0.001 vs. control, ### p<0.001 vs. HG, ††† p<0.001 vs. HG+RESV. **(G and H)** HG-induced increase in TGF-β1 protein expression. ***p< 0.001 vs. control, ### p< 0.001 vs. HG (One-way ANOVA p< 0.05). **(I and J)** Vascular smooth muscle cells were pre-treated with resveratrol for 1 hr, followed by stimulation with HG and exogenously added recombinant TGF-β1. Protein expression levels of collagen type I alpha 1 and DDR2 were examined at 12 h by western blot analysis. *** p<0.001 vs. control, ### p<0.001 vs. HG, ††† p<0.001 vs. HG+RESV (For DDR2 and collagen type 1 alpha 1). (One-way ANOVA p< 0.05). Data are representative of three independent experiments with SD, n = 3. Error bars represent SD.

To check whether similar mechanisms exist in VSMCs, cells were exposed to hyperglycemic conditions and DDR2 and collagen type I alpha 1 mRNA and protein expression patterns were analyzed. Significant up-regulation of collagen type I and DDR2 expression (Fig 6A–6F) was observed in VSMCs as well. Moreover, the effect of HG on collagen type I and DDR2 expression was sustained at 48 h (S4C and S4D Fig). Interestingly, the regulatory relationship between DDR2 and collagen type I seen in adventitial fibroblasts was observed in VSMCs exposed to HG (Fig 6G and 6H) and under basal conditions (S4F Fig). Further, as in adventitial fibroblasts, resveratrol (50 μM) abolished HG-induced increase in collagen type I alpha 1 (Fig 7A and 7B) and DDR2 in VSMCs (Fig 7C and 7D). Resveratrol also attenuated the basal expression levels of collagen type I alpha 1 and DDR2 in VSMCs (S4K and S4L Fig). While resveratrol attenuated HG-stimulated collagen type I expression in VSMCs, the effect was not observed in DDR2-overexpressing cells, showing that attenuation of HG-stimulated collagen type I expression by resveratrol is due to DDR2 inhibition (Fig 7E and 7F). Moreover, while HG-induced TGF-β expression in VSMCs was reduced by resveratrol (Fig 7G and 7H), exogenous addition of recombinant TGF-β-1 (10 ng/mL) restored DDR2 and collagen type I alpha 1 expression in resveratrol-treated cells, suggesting that resveratrol inhibits TGF-β1 to downregulate DDR2 and collagen type I expression in HG-treated cells (Fig 7I and 7J).

#### Association of DDR2 with collagen deposition and remodeling in the abdominal aorta in a monkey model of metabolic syndrome

**Fig 8:**
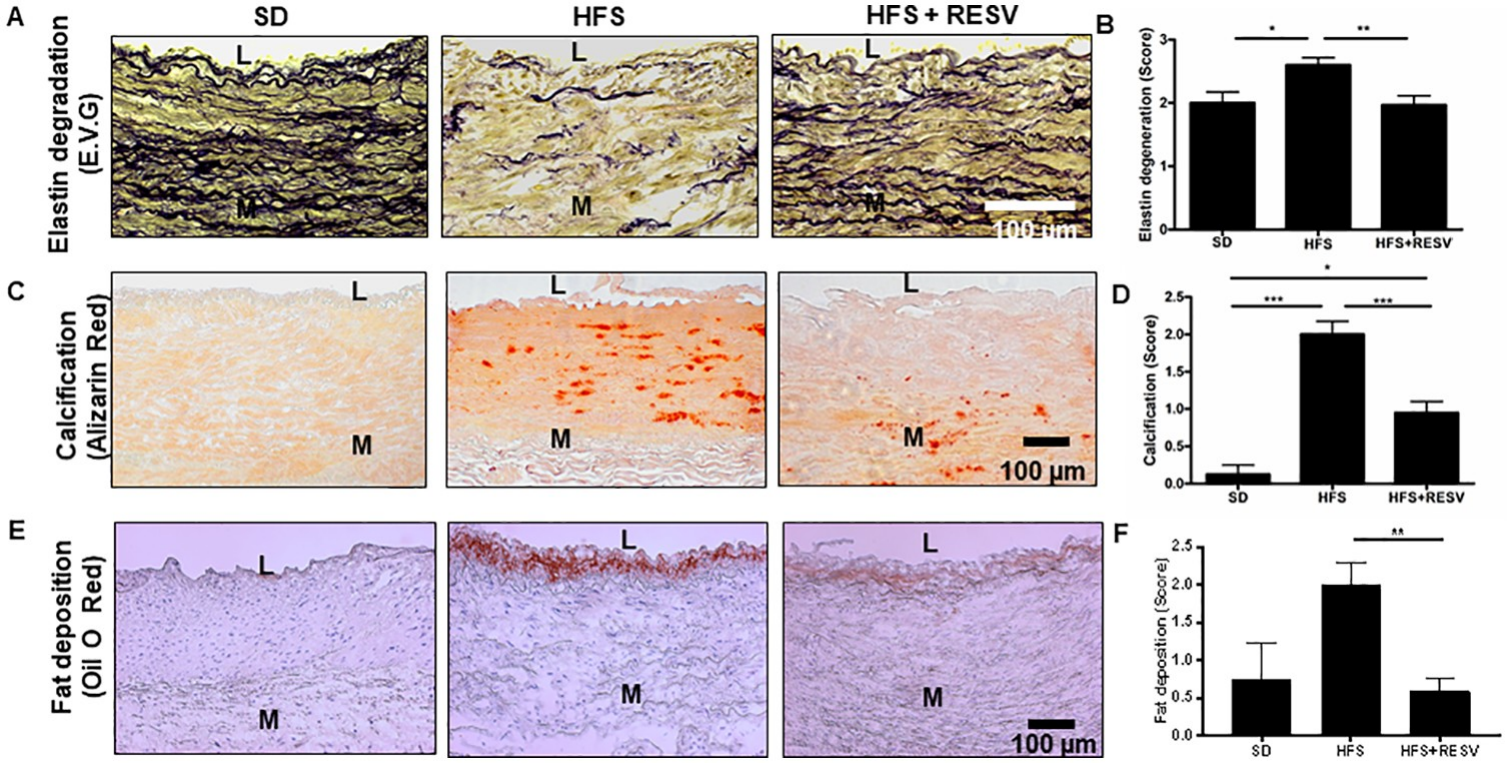
HFS-induced abdominal aortic remodeling in a monkey model of metabolic syndrome. **(A)** Representative photomicrographs of EVG staining of paraffin sections (5 μm) demonstrating abdominal aortic elastin laminae degradation/erosion in rhesus monkeys. **(B)** Data of the scoring of elastin degradation (4–6 consecutive cross sections) in SD (n = 4), HFS (n = 10), and HFS+RESV (n = 10) monkeys presented as Mean ± SEM. One-way ANOVA Kruskal-Wallis with Post hoc Dunn’s test, *p<0.05 vs. SD, **p<0.01 vs. HFS. **(C)** Representative photomicrographs of Alizarin red staining of frozen sections (6 μm) demonstrating abdominal aortic calcification. **(D)** Data of the scoring of calcium deposits (4–6 consecutive cross sections) in SD (n = 4), HFS (n = 10), and HFS+RESV (n = 10) presented as Mean ± SEM. One-way ANOVA Kruskal-Wallis with Post hoc Dunn’s test, *p<0.05 vs. SD, ***p<0.001 vs. SD. **(E)** Representative photomicrographs of oil O red staining of frozen sections (6 μm) demonstrating abdominal aortic fat deposition in rhesus monkeys. **(F)** Data scoring of fat deposits (4 consecutive cross sections) in SD(n = 4), HFS(n = 10), and HFS+RESV(n = 10) presented as Mean ± SEM. One-way ANOVA Kruskal-Wallis with Post hoc Dunn’s test, **p<0.01 vs. HFS. SD = Standard Diet, HFS = High Fat Sucrose Diet, HFS+RESV = High Fat Sucrose Diet with resveratrol treatment. L = Vascular Lumen, M = Vascular medium.

**Fig 9:**
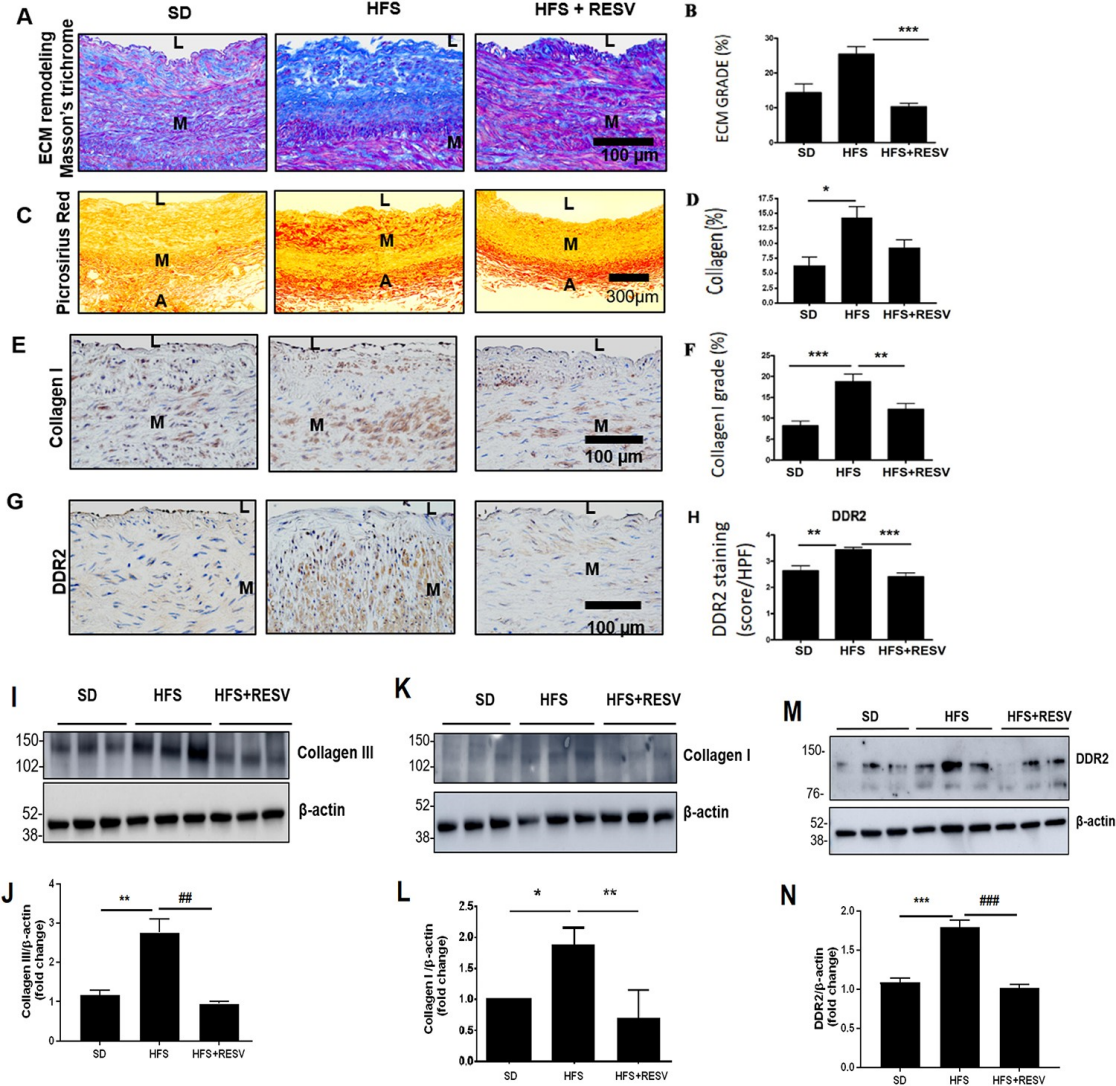
HFS-induced alterations in ECM, collagen, and DDR2 in the abdominal aortic wall. **(A)** Representative photomicrographs of Masson's trichrome staining of paraffin sections (5 μm) demonstrating extracellular matrix (blue) deposition in the abdominal aorta of rhesus monkeys. **(B)** Percentage increase in ECM remodeling in SD (n = 4), HFS (n = 10), and HFS+RESV (n = 10) presented as Mean ± SEM. 4 consecutive cross sections were analyzed. One-way ANOVA Kruskal-Wallis with Post hoc Dunn’s test, ***p<0.001 vs. HFS. **(C)** Representative photomicrographs of Sirius red staining of paraffin sections (5 μm) that demonstrate total collagen in the abdominal aorta of rhesus monkeys. **(D)** Percentage increase in total collagen in SD (n = 4), HFS (n = 10), and HFS+RESV (n = 10) presented as Mean ± SEM. 4 consecutive cross sections were analyzed. One-way ANOVA Kruskal-Wallis with Post hoc Dunn’s test, *p<0.05. **(E)** Representative photomicrographs of type I collagen immunostaining of paraffin sections (5 μm) that demonstrate collagen type I deposition in the abdominal aorta of rhesus monkeys. **(F)** Percentage increase in collagen type I in SD (n = 4), HFS (n = 10), and HFS+RESV (n = 10) presented as Mean ± SEM. 4 consecutive cross sections were analyzed. One-way ANOVA Kruskal-Wallis with Post hoc Dunn’s test. ***p<0.001 vs. SD, **p<0.01 vs. HFS. **(G)** Representative photomicrographs of DDR2 immunostaining (brown) of frozen sections (5 μm) demonstrating DDR2 expression in the abdominal aorta of rhesus monkeys. **(H)** Data of the scoring of DDR2 protein signal in SD (n = 4), HFS (n = 10), and HFS+RESV (n = 10) presented as Mean ± SEM. 4–6 consecutive cross sections were analyzed. One-way ANOVA Kruskal-Wallis with Post hoc Dunn’s test **p<0.01 vs. SD, ***p<0.001 vs. HFS. **(I-N)** Expression levels of collagen types I and III, and DDR2, in the abdominal aorta from SD, HFS and HFS+RESV-fed rhesus monkeys were analyzed by western blotting. For collagen type III—** p< 0.01 vs. SD, ## p< 0.01 vs. HFS. For collagen type I—* p< 0.05 vs. SD, ** p< 0.01 vs. HFS. For DDR2—*** p< 0.001 vs. SD, ### p<0.001 vs. HFS. (One-way ANOVA p< 0.05). Data are representative of 3 independent experiments with SD, n = 3. Error bars represent SD.SD = Standard Diet, HFS = High Fat Sucrose Diet, HFS+RESV = High Fat Sucrose Diet with resveratrol treatment. L = Vascular Lumen, M = Vascular medium, A = Vascular Adventitia.

A significant increase in body weight and cholesterol, loss of endothelial cell integrity, lipid and macrophage infiltration and elevated blood pressure were earlier reported by us in adult male *Macaca mulatta* fed a HFS diet for 2 years [21]. In the present study, the HFS diet was found to induce adverse abdominal aortic remodeling, as evidenced by extensive degradation of the elastic laminae (Fig 8A and 8B), calcification (Fig 8C and 8D) enhanced staining for fat deposition (Fig 8E and 8F), and ECM remodeling events (Fig 9A–9F).

**Fig 10:**
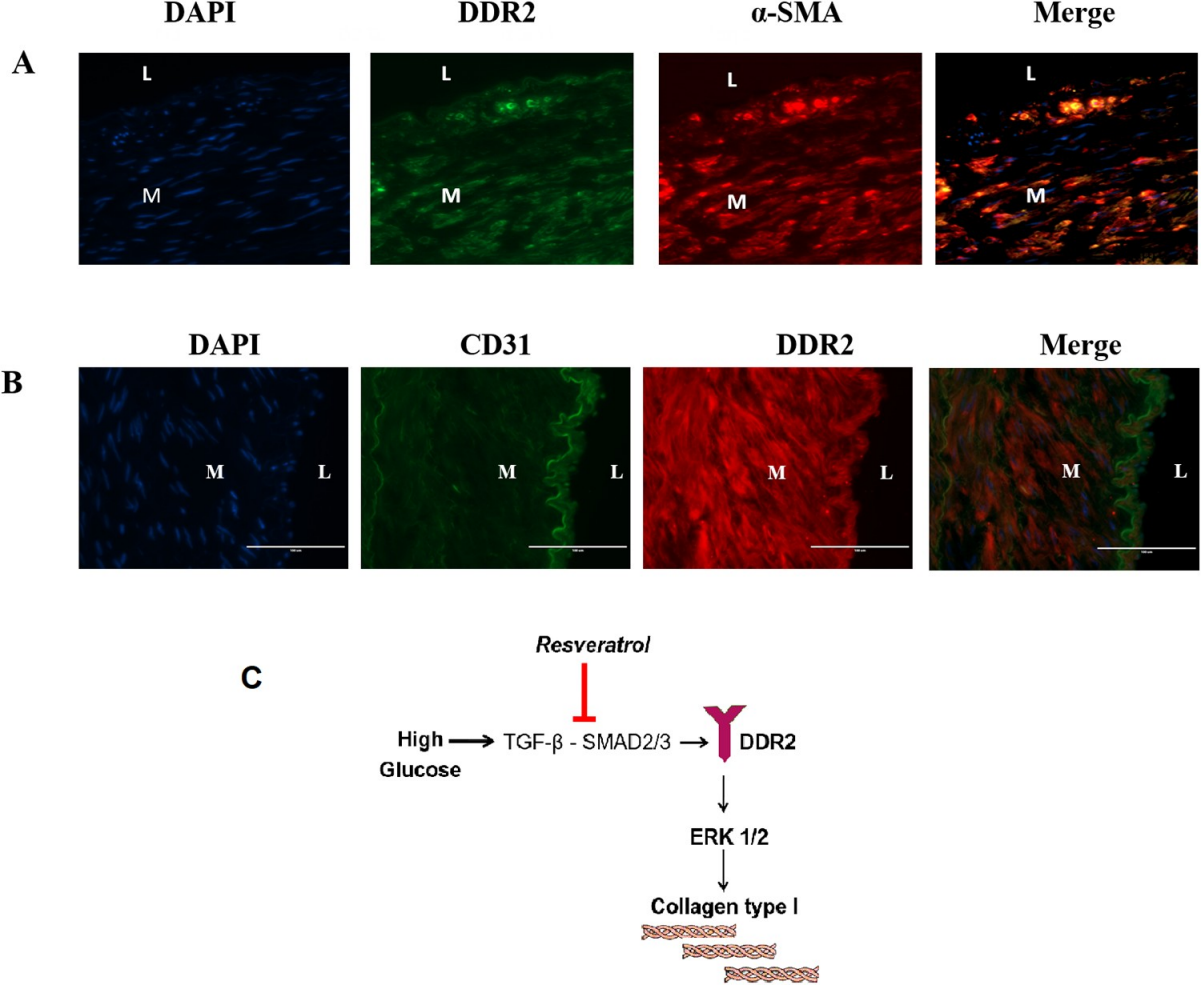
Localization of DDR2 with markers of fibroblasts, VSMCs and endothelial cells in the abdominal aortic wall. **(A)** Dual labeling of abdominal aortic tissue sections for DDR2 and α-SMA. Nuclei were counter-stained with DAPI (blue). **(B)** Dual labeling of abdominal aortic tissue sections for endothelial cell marker-CD31 (green) and DDR2 **(C)** Schematic representation of DDR2-dependent increase in collagen type 1 gene expression in vascular cells exposed to hyperglycemic conditions.

A significant increase in ECM remodeling (Fig 9A and 9B) along with total collagen levels (Fig 9C and 9D) was observed in the abdominal aortic wall in animals fed the HFS diet for 2 years. Importantly, the changes in collagen type I expression levels (Fig 9E and 9F) and total collagen levels (Fig 9C and 9D) were associated with a marked increase in DDR2 in the arterial wall in animals fed the HFS diet (Fig 9G and 9H). The study explored the effect of resveratrol on HFS diet-induced changes in the animals. A significant reduction in the markers of arterial remodeling and atherogenic progression, including elastin degradation, calcification and fat deposition, was observed in the resveratrol-fed animals (Fig 8A–8F). Resveratrol also produced a marked decrease in DDR2 expression that correlated with a reduction in ECM remodeling, collagen remodeling and collagen type I immunostaining in these animals (Fig 9A–9H). Consistent with these observations, western blot analysis showed a significant increase in the expression of collagen types I and III in the abdominal aortic tissue from the HFS-fed group, which correlated with an increase in the expression of DDR2. These changes were not observed in the resveratrol-group (Fig 9I–9N). The colocalization of DDR2 in adventitial fibroblasts, VSMCs and endothelial cells was analyzed (Fig 10A and 10B). DDR2 was found to colocalize with α-SMA in fibroblasts and VSMCs (Fig 10A). Colocalization of DDR2 was not observed in endothelial cells (Fig 10B). Fig 10C provides a schematic representation of DDR2-dependent increase in collagen type 1 gene expression in vascular cells exposed to hyperglycemic conditions.

## Discussion

Metabolic syndrome, which is a cluster of conditions including elevated blood pressure, high blood sugar, dyslipdemia and abdominal obesity, is a significant contributor to the pathogenesis of vascular fibrosis[23]. Risk factors associated with metabolic syndrome alter the phenotype of adventitial fibroblasts and VSMCs in favor of enhanced collagen production to promote vascular fibrosis and its sequelae[18]. The underlying mechanisms, however, remain largely unexplored.

Our earlier studies demonstrated that HFS diet induces a marked increase in central arterial stiffening, as evidenced by an increase in pulse wave velocity, and enhanced signatures of inflammatory markers driven by oxidative stress in the thoracic aorta of adult male rhesus monkeys[21]. The present study employed this model of metabolic stress to focus on ECM remodeling in the abdominal aortic wall, which is prone to atherosclerotic remodeling in response to metabolic insult. Here, we show a significant increase in collagen type I deposition within the medial and adventitial layers and adverse collagen remodeling in the HFS-fed group (Fig 9A–9F). Notably, the increase in collagen deposition and adverse ECM remodeling correlated well with increased expression of DDR2 within adventitial fibroblasts and VSMCs in the HFS-fed group (Fig 9G and 9H), suggesting a possible link between DDR2 and collagen production in a setting of metabolic syndrome. Further, the present investigations on isolated vascular cells indicated an obligate role for DDR2 in collagen type I gene expression in adventitial fibroblasts and VSMCs exposed in vitro to hyperglycemia, which is an important risk factor associated with metabolic syndrome. The finding is in accord with a previous report from our laboratory that Angiotensin II-stimulated collagen type I expression in cardiac fibroblasts is DDR2-dependent[24]. Together, the findings point to the shared characteristic of a regulatory relationship between DDR2 and collagen in cells of mesenchymal origin that express both DDR2 and collagen, which may be an important determinant of fibrosis in the cardiovascular system.

Degeneration of compliant elastin fibers, and deposition of stiffer collagen, is considered an important cause of arterial stiffening. The HFS diet was found to induce elastin degradation in the arterial wall, which can contribute significantly to loss of arterial resilience and consequent increase in arterial stiffness. In this regard, DDR2 is known to trigger matrix metalloproteinase-2 activation[18], which in turn is closely associated with elastolysis, calcification and atherosclerotic plaque vulnerability, the hallmark of metabolic arterial syndrome [25]. It is likely that, in addition to promoting collagen deposition and remodeling, DDR2 may also have a role in inducing other structural changes in the arterial wall via MMP-2 activation.

It is pertinent to note that previous studies on DDRs in relation to collagen remodeling during vascular injury and progression of atherosclerosis were focused largely on DDR1. For example, Hou et al. (2001) demonstrated a positive regulatory role for DDR1 in collagen expression and deposition in a rat model of carotid artery injury[26]. However, another study by the same group reported a negative correlation between DDR1 and collagen production within plaques of atherosclerotic mice, which may contribute to increased plaque stability[27]. The study also demonstrated an increase in pro-collagen type I and III mRNA expression in VSMCs lacking DDR1, indicating an inverse relationship between DDR1 and collagen. On the other hand, a link between DDR2 and vascular fibrosis has not hitherto been demonstrated although the regulation of DDR2 in vascular cells has been addressed earlier[28]. A study by Ferri et al. (2004) on atherosclerosis-associated collagen remodeling in non-human primate models showed that early and late stage atherosclerotic plaques express DDR1 and DDR2[29]. The study did not correlate DDR2 levels with collagen levels. However, based on the observation that over-expression of DDR1 or DDR2 in isolated VSMCs plated on polymerized collagen-coated dishes down-regulated collagen mRNA expression, the authors concluded that DDRs may promote plaque instability. Furthermore, another study[30] found no significant difference in basal levels of expression of collagen type I in VSMCs isolated from DDR2 knockout mice, compared to the wild-type, which could be due to compensatory mechanisms in the knockout model. In contrast to these reports, our findings from both the in vitro and in vivo models point to a positive correlation between DDR2 and collagen expression and remodeling, suggestive of a role for DDR2 in promoting arterial fibrosis associated with metabolic syndrome.

Our in vitro studies demonstrate the effect of hyperglycemia on collagen production, which is generally attributed to the action of Angiotensin II[31] and TGF-β1 [32]. We found that TGF-β1/SMAD2/3 signaling mediates the stimulatory effect of hyperglycemia on DDR2 that in turn enhances collagen type 1 expression via ERK1/2 MAPK activation (Figs 2–4). Notably, while TGF-β1 knockdown inhibited DDR2 and collagen expression, the effect of TGF-β1 inhibition on collagen expression was not observed in cells over-expressing DDR2 (Fig 2E–2H), clearly demonstrating the centrality of DDR2 in mediating TGF-β1-dependent collagen type I expression in these cells. Together, the data presented here suggest that DDR2 may be a key player in the progression of vascular fibrosis and an early marker of fibrotic vascular remodeling. In this context, it is pertinent to note that DDR2 has previously been linked to pulmonary fibrosis in mice [24].

Interestingly, Mattison et al. (2014) had earlier demonstrated the therapeutic benefits of resveratrol in preventing arterial wall stiffening through amelioration of vascular inflammation and oxidative stress in this model of metabolic syndrome[21]. Resveratrol is reported to affect several pathways in various disease models [33,34]. Although it has been shown to reduce collagen production in VSMCs[35] and in rodent models of fibrosis[36], neither its molecular target nor its beneficial effect in mitigating vascular fibrosis through down-regulation of collagen production has hitherto been demonstrated in a clinically-relevant model of metabolic syndrome. In the present study, resveratrol addition to the HFS diet reduced elastin degradation and markers of adverse arterial remodeling such as calcification and fat deposition within the arterial wall. Further, dietary supplementation of resveratrol attenuated the increase in collagen type I and adverse collagen remodeling in the vascular wall. Importantly, the reduction in collagen content and ECM remodeling was associated with a concomitant reduction in DDR2 within the aortic adventitia and media, raising the possibility that resveratrol may exert its beneficial effects via DDR2. Such a postulation is supported by our observations that resveratrol abolishes the stimulatory effect of hyperglycemia on DDR2 and collagen in isolated adventitial fibroblasts and VSMCs (Figs 5A–5D and 7A–7D). Notably, while resveratrol attenuated HG-stimulated collagen expression, the effect was not observed in DDR2-overexpressing cells (Figs 5G, 5H, 7E and 7F). Considered in tandem, these observations suggest that DDR2 could be a target of resveratrol in ameliorating vascular fibrosis.

In summary, the findings from this study support a role for DDR2 in the pathogenesis of vascular fibrosis in a setting of metabolic syndrome. Since vascular fibroblasts and VSMCs are the predominant source of both DDR2 and collagen in the vasculature, the regulatory relationship between DDR2 and collagen in these cell types underscores the existence of a molecular circuitry involving these two pro-fibrotic factors that may drive tissue fibrosis in response to injury. Clearly, with its specific localization in adventitial fibroblasts and VSMCs in the vascular wall, DDR2 emerges as a potential drug target in the management of adverse fibrotic vascular remodeling.

## Supporting information

S1 Fig Characterization of adventitial fibroblasts and VSMCs. (DOCX)

S2 Fig Characterization of adventitial fibroblasts and VSMCs. (DOCX)

S3 Fig Analysis of overexpression efficiency in adventitial fibroblasts and VSMCs. (DOCX)

S4 Fig Basal expression levels of protein. (DOCX)

S5 Fig Full blots of the representative images. (DOCX)
