# Supplementary material for: Discoidin domain Receptor 2: A determinant of metabolic syndrome-associated arterial fibrosis in non-human primates
Source: PLoS One. 2019 Dec 5;14(12):e0225911. doi: 10.1371/journal.pone.0225911 (PMC6894805; doi:10.1371/journal.pone.0225911)
Supplement: S1 Fig — (DOCX) [file pone.0225911.s001.docx]

##
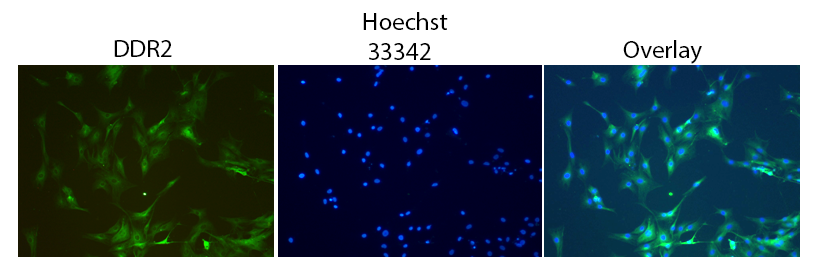


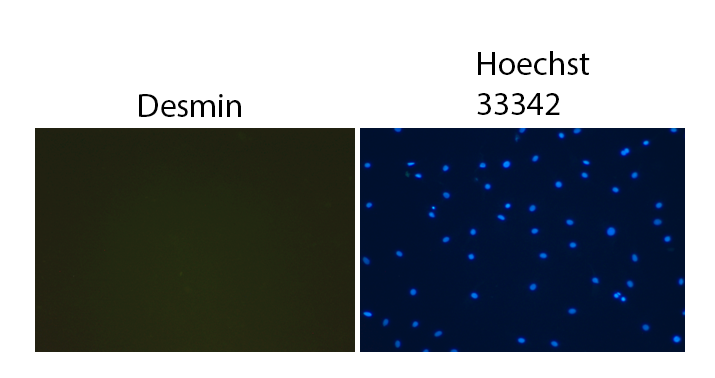


## Figure S1 : Sub-confluent cultures were immunostained with anti-DDR2 antibody and anti-Desmin antibody respectively. Nuclei were counter-stained with Hoechst 33342. 10X magnification
