# Supplementary material for: Discoidin domain Receptor 2: A determinant of metabolic syndrome-associated arterial fibrosis in non-human primates
Source: PLoS One. 2019 Dec 5;14(12):e0225911. doi: 10.1371/journal.pone.0225911 (PMC6894805; doi:10.1371/journal.pone.0225911)
Supplement: S2 Fig — (DOCX) [file pone.0225911.s002.docx]

**
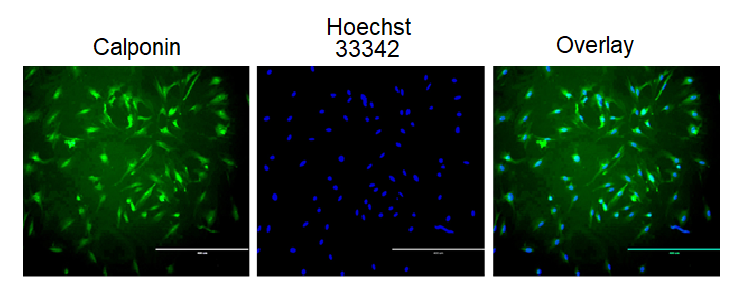
**

## Figure S2: Sub-confluent cultures of VSMC’s were immunostained with anti-Calponin antibody . Nuclei were counter-stained with Hoechst 33342. 10X magnification.
