# Supplementary material for: Discoidin domain Receptor 2: A determinant of metabolic syndrome-associated arterial fibrosis in non-human primates
Source: PLoS One. 2019 Dec 5;14(12):e0225911. doi: 10.1371/journal.pone.0225911 (PMC6894805; doi:10.1371/journal.pone.0225911)
Supplement: S3 Fig — (DOCX) [file pone.0225911.s003.docx]

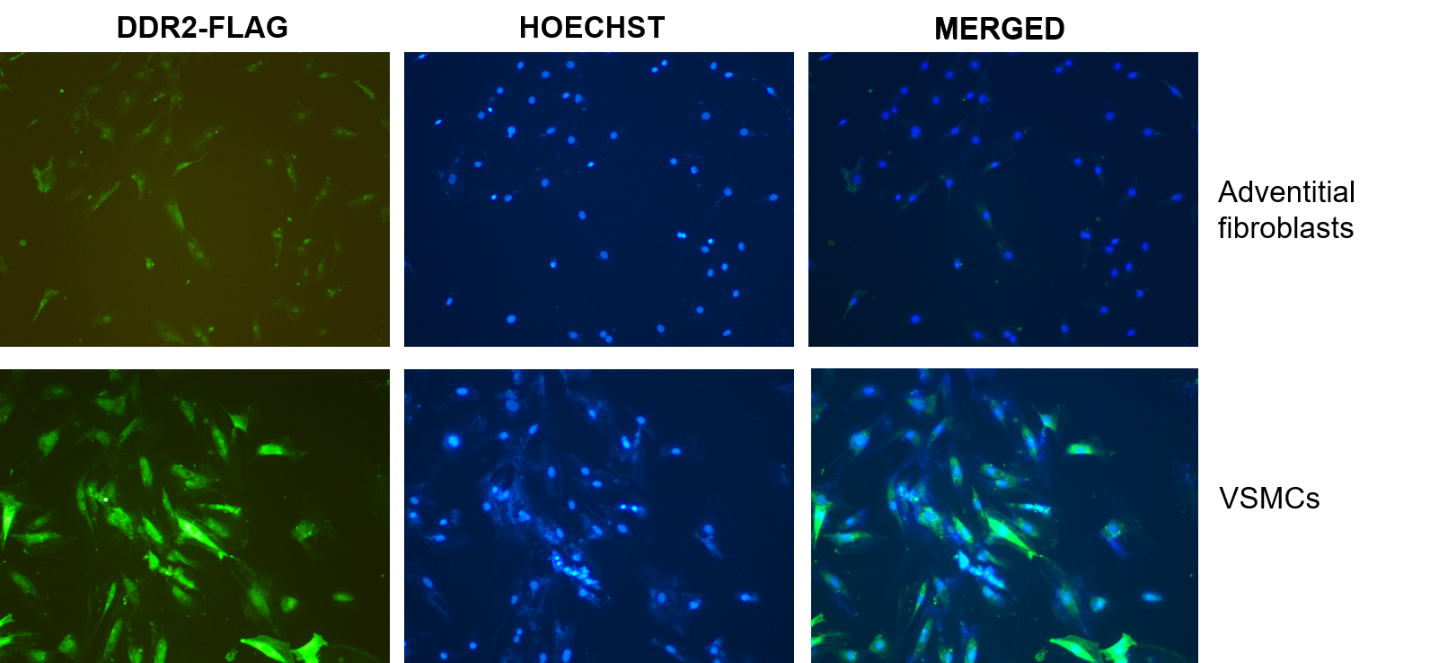


**Supplementary Fig. S3:** Vascular adventitial fibroblasts or VSMCs were transiently transfected with FLAG-tagged DDR2 overexpression vector. The overexpression efficiency was in (A) adventitial fibroblasts and (B) VSMCs were analyzed. 4-5 fields were analyzed.
