# Supplementary material for: Discoidin domain Receptor 2: A determinant of metabolic syndrome-associated arterial fibrosis in non-human primates
Source: PLoS One. 2019 Dec 5;14(12):e0225911. doi: 10.1371/journal.pone.0225911 (PMC6894805; doi:10.1371/journal.pone.0225911)
Supplement: S4 Fig — (DOCX) [file pone.0225911.s004.docx]

**
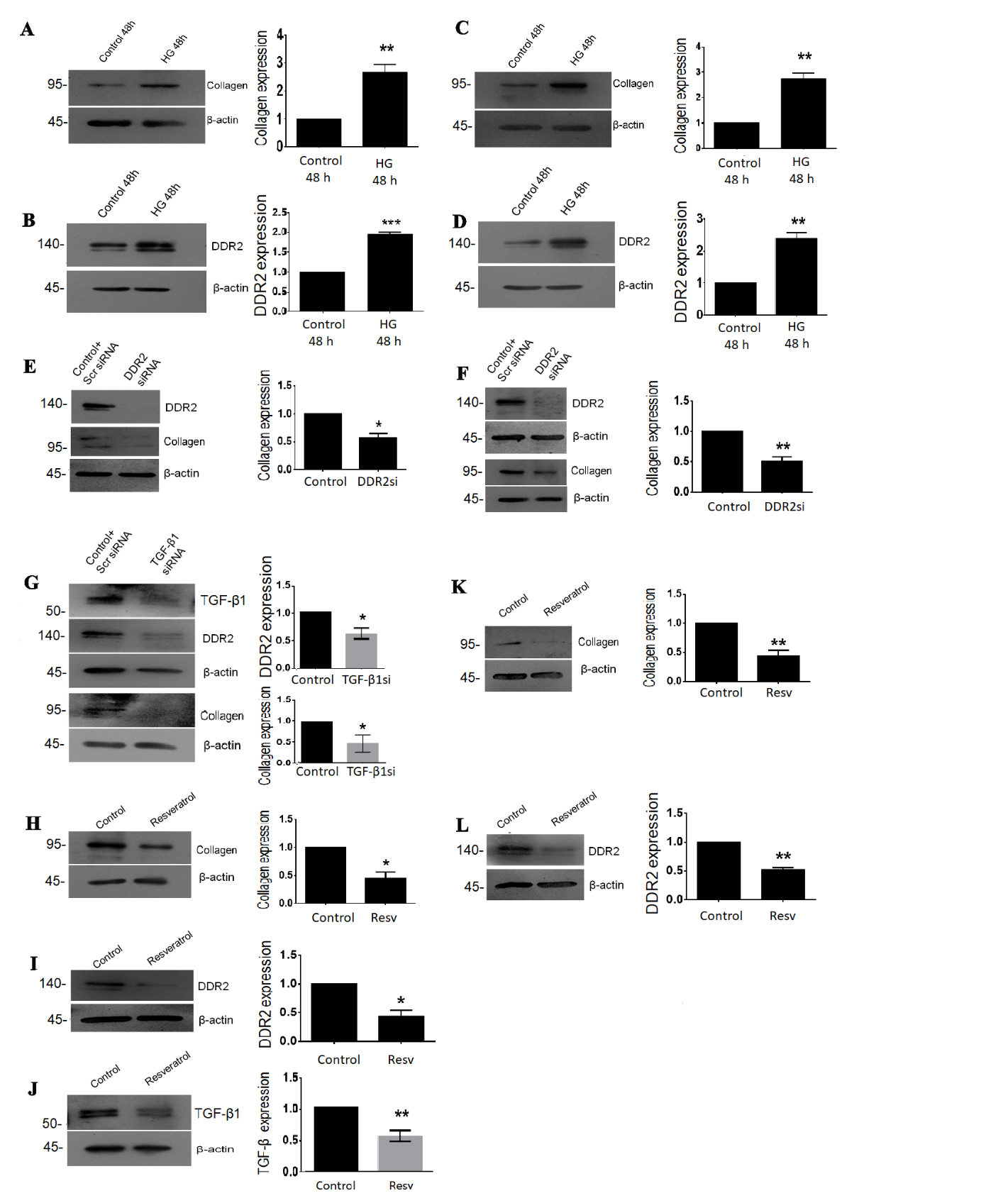
**

**Supplementary Fig.S4 :** Chronic exposure to HG stimulates collagen type I and DDR2 expression in **(A,B)** vascular adventitial fibroblasts and **(C,D)** VSMCs. Sub-confluent quiescent cultures of vascular adventitial fibroblasts and VSMCs respectively in M199 were treated with 25mM HG for 48 h. Protein was isolated and subjected to western blot analysis for detection ofcollagen type I and DDR2, with β-actin as loading control. **p< 0.01 vs. control, *** p< 0.001 vs. HG (paired, two-tailed Student’s *t*-test). **(E)**Regulatory relationship between DDR2 and collagen type I in vascular adventitial fibroblasts. Vascular adventitial fibroblasts were transiently transfected with DDR2 siRNA. Protein was isolated at 12 h and subjected to western blot analysis for detection of DDR2 and collagen type I, with β-actin as loading control. *p< 0.05 vs. control, (paired, two-tailed Student’s *t*-test).**(F)**Regulatory relationship between DDR2 and collagen type I in VSMCs. VSMCs were transiently transfected with DDR2siRNA. Protein was isolated at 12 h and subjected to western blot analysis for detection of DDR2 and collagen type I, with β-actin as loading control. **p< 0.01 vs. control, (paired, two-tailed Student’s *t*-test).**(G)** Knockdown of TGF-β1 attenuated basal expression levels of DDR2 and collagen type I in vascular adventitial fibroblasts. Vascular adventitial fibroblasts were transiently transfected with TGF-β1 siRNA. Protein was isolated at 12 h and subjected to western blot analysis for detection ofTGF-β1, DDR2 and collagen type I, with β-actin as loading control. *p< 0.05 vs. control, (paired, two-tailed Student’s *t*-test). Resveratrol attenuated basal expression levels of **(H)**collagen type I**(I)**DDR2 and **(J)** TGF-β1 in vascular adventitial fibroblasts.Sub-confluent quiescent cultures of vascular adventitial fibroblasts in M199 were treated with Resveratrol (25µM).Protein was isolated at 12 h post-treatment and subjected to western blot analysis for detection of collagen type I, DDR2 and TGF-β1 with β-actin as loading control. *p< 0.05 vs. control,**p< 0.01 vs. control (paired, two-tailed Student’s *t*-test).Resveratrol attenuated basal expression levels of **(K)**collagen type I and **(L)**DDR2 in VSMCs. Sub-confluent quiescent cultures of VSMCs in M199 were treated with Resveratrol (25µM). Protein was isolated at 12 h post-treatment and subjected to western blot analysis for detection of collagen type I and DDR2, with β-actin as loading control. **p< 0.01 vs. control (paired, two-tailed Student’s *t*-test).
