# Supplementary material for: Discoidin domain Receptor 2: A determinant of metabolic syndrome-associated arterial fibrosis in non-human primates
Source: PLoS One. 2019 Dec 5;14(12):e0225911. doi: 10.1371/journal.pone.0225911 (PMC6894805; doi:10.1371/journal.pone.0225911)

**Supplementary Fig. S5 :** Full length blots corresponding to the main figures in the article text and supplementary Fig. S3

**HG enhances collagen type I and DDR2 gene expression in vascular adventitial fibroblasts.**Uncropped blots corresponding to Fig.1 in the main article text. Boxed regions are used in the main figures.

1. Collagen with the corresponding β-actin for Fig 1 B

**
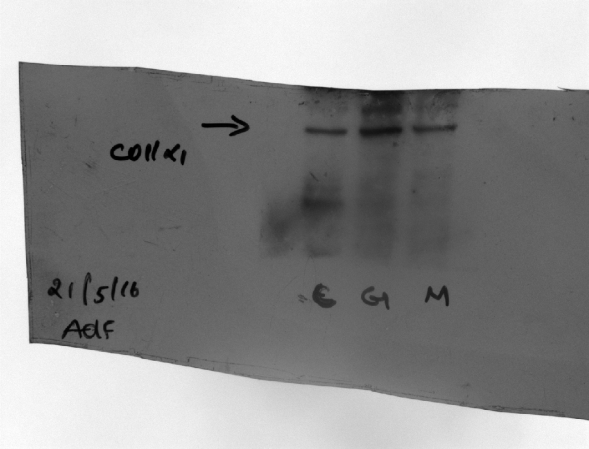

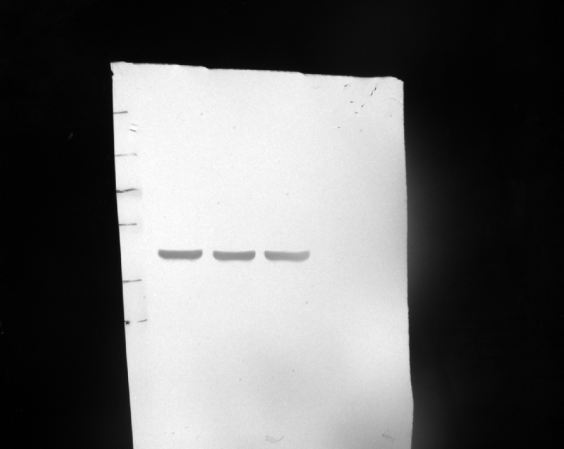
**

**B.** DDR2 with the corresponding β-actinFig 1 E

**
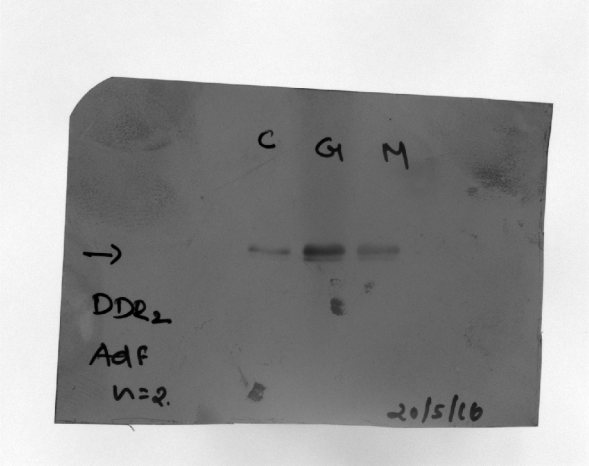

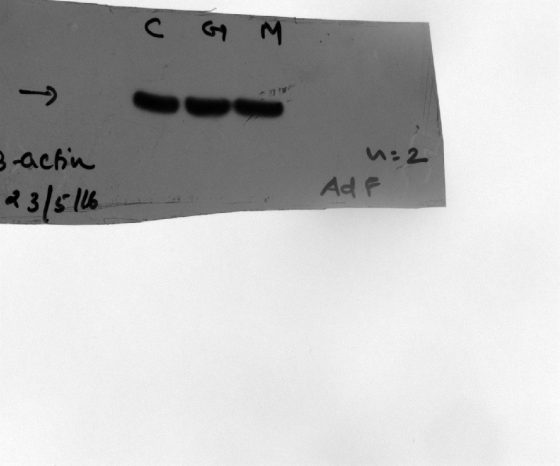
**

**C**

1. DDR2 siRNA validation along with Collagen and the corresponding β-actin for Fig. 1 G

Collagen

DDR2 siRNA validation


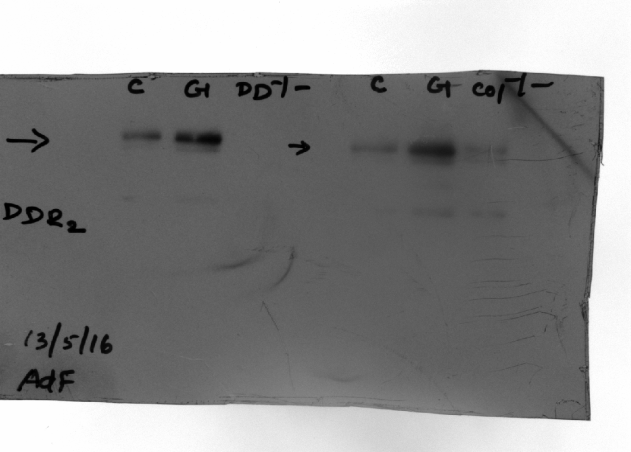

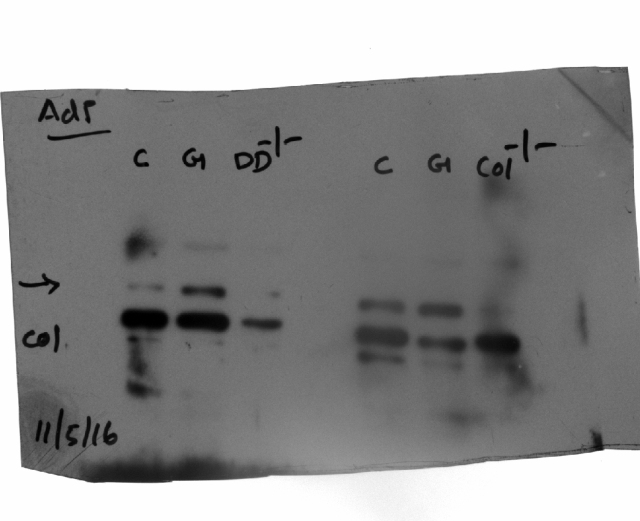


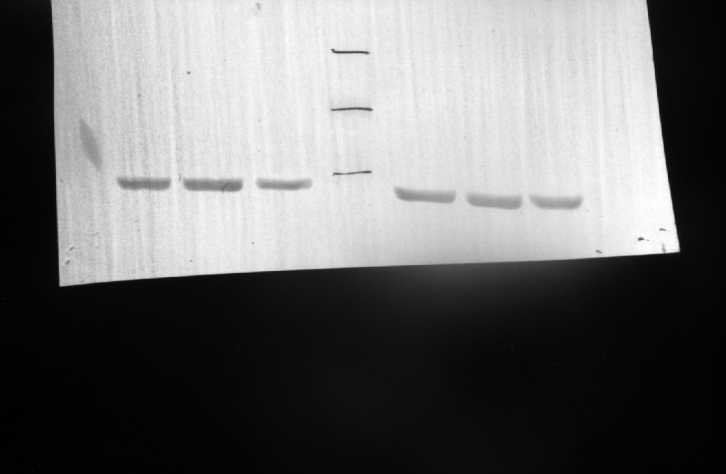


β-actin

Control HG HG+DDR2siRNA

**TGF-β1 mediates HG-induced DDR2 and collagen gene expression.**Uncropped blots corresponding to Fig.2 in the main article text. Boxed regions are used in the main figures.

a. Collagen with the corresponding β-actin for Fig. 2 A


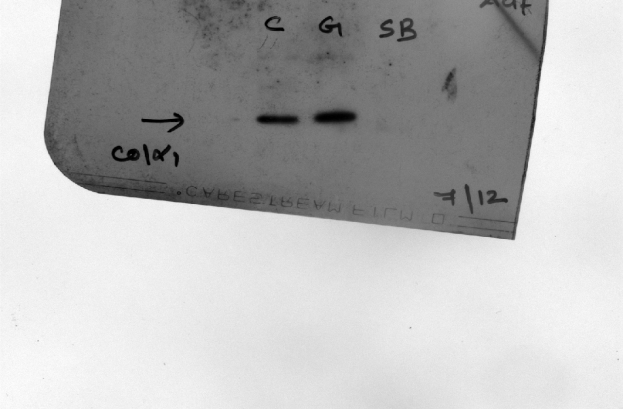

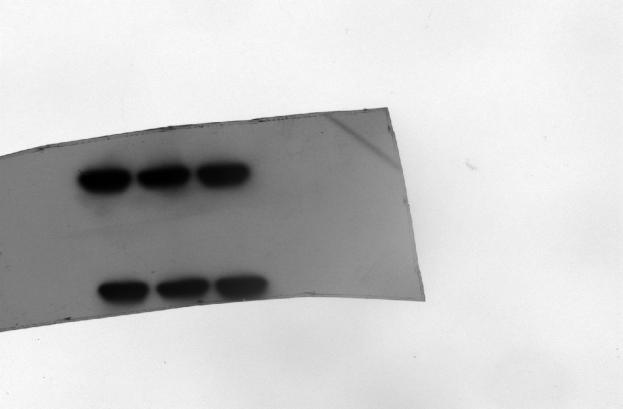


Multiple exposures of β-actin

b. DDR2 with the corresponding β-actin for Fig. 2 C


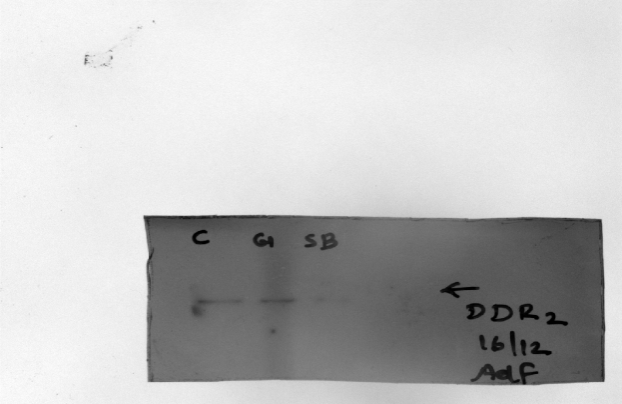

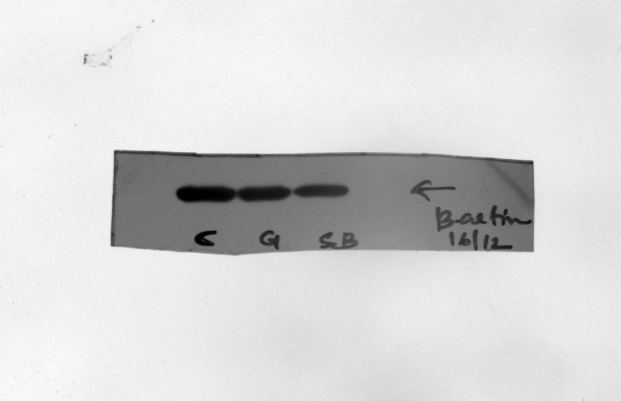


1. TGF-β siRNA validation blots with the corresponding DDR2, Collagen and β-actin for Fig. 2 E

**
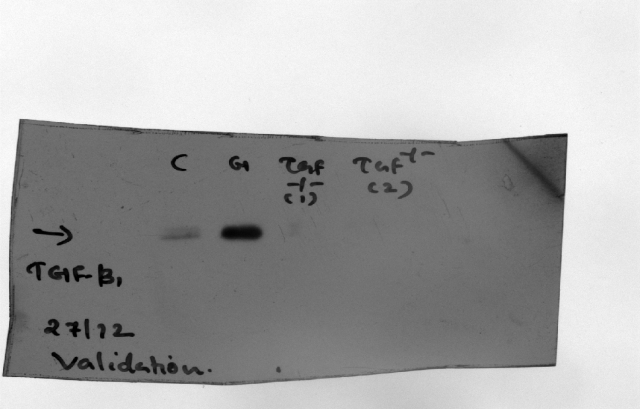
**

TGF-β siRNA validation

**
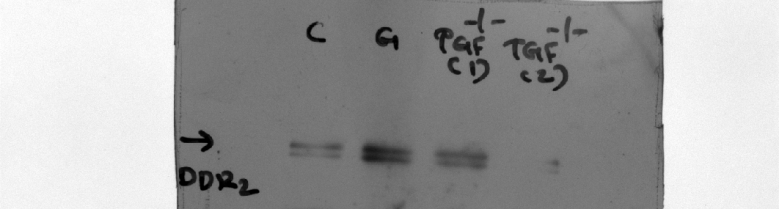
**

DDR2

**
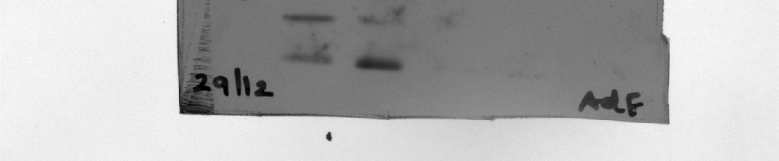
**

Collagen

**
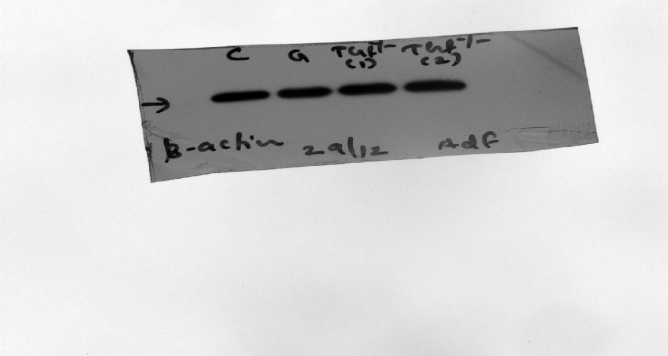
**

β-actin

**C.** DDR2 and Collagen blots with the corresponding β-actin for Fig. 2 G

**
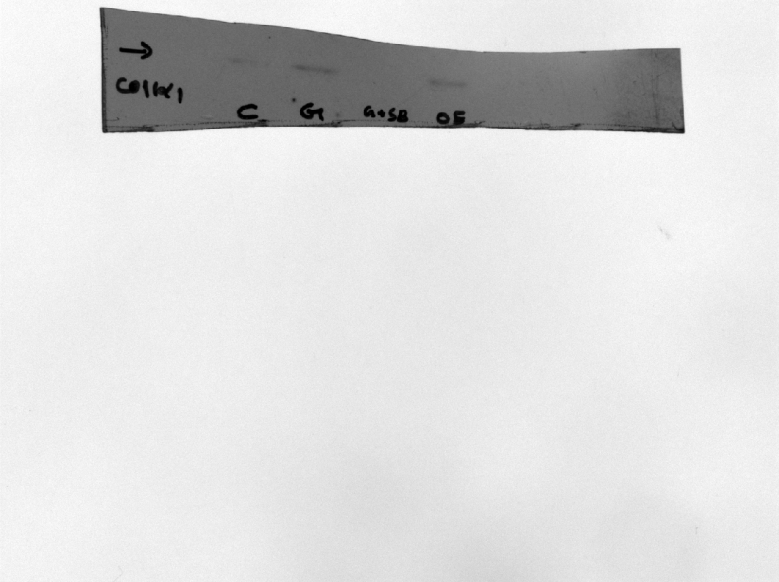
**

Collagen representative blot

**
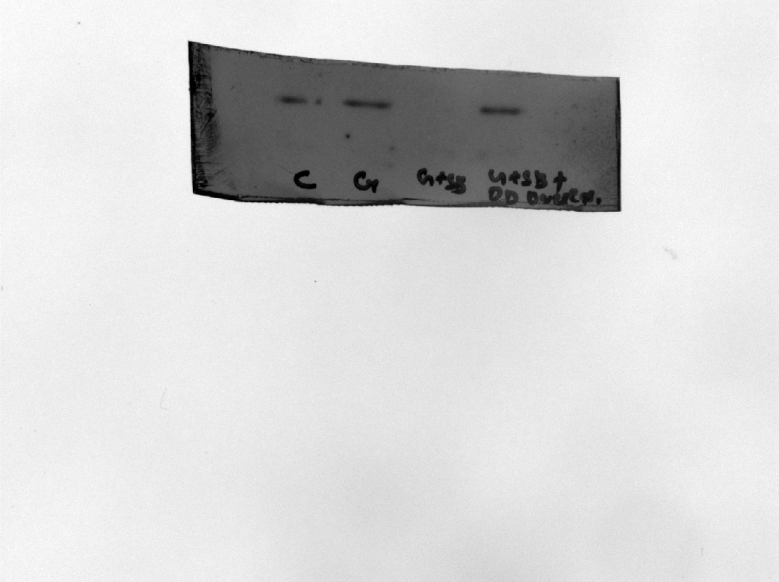
**

Collagen blot 2^nd^ exposure

**
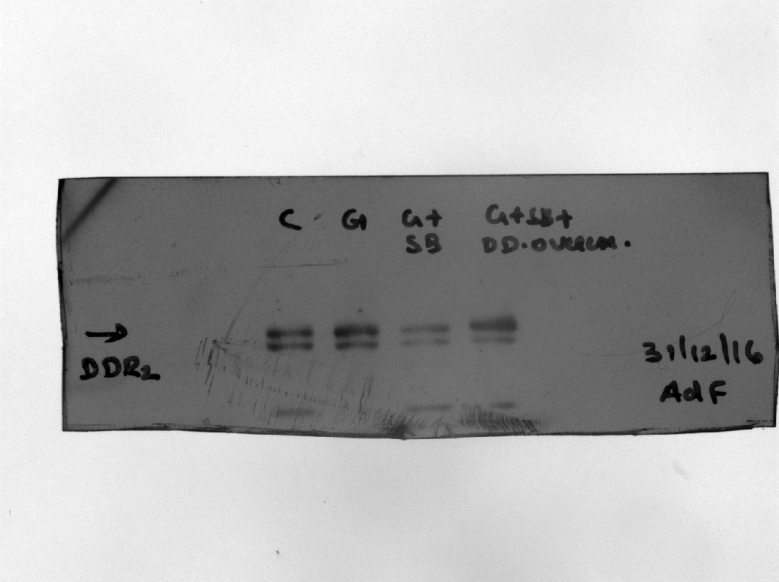
**

DDR2 exposure 1

**
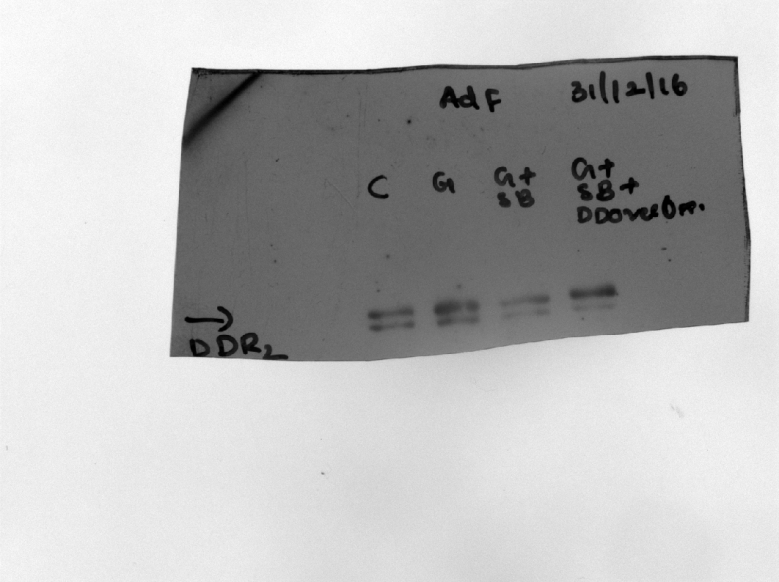
**

DDR2 exposure 2

**
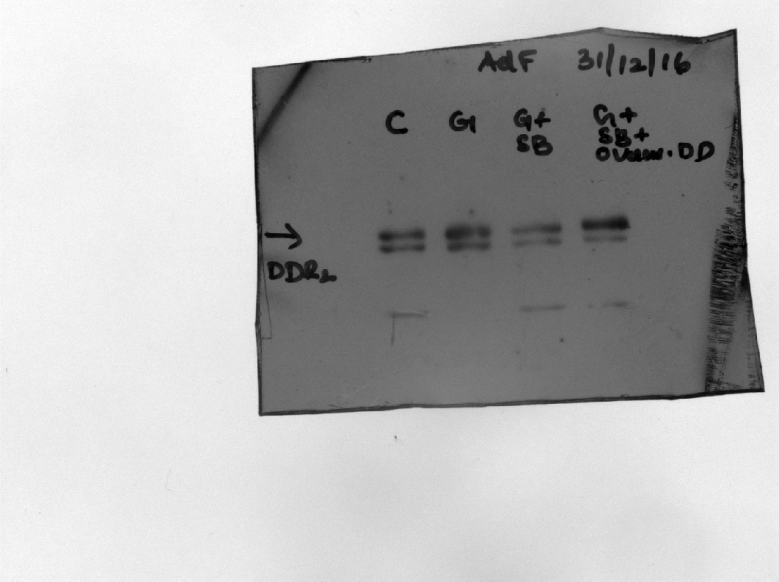

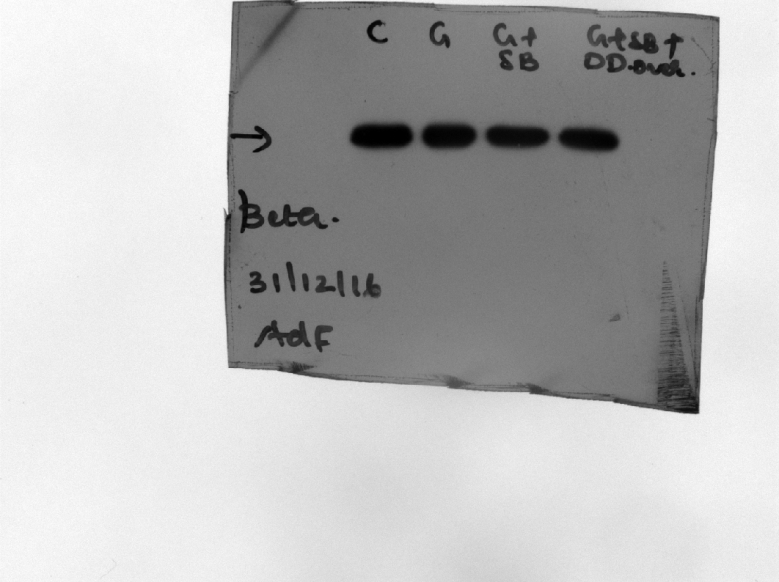
**

DDR2 exposure 3

β-actin

**SMAD2/3 inhibition attenuates HG-induced DDR2 and collagen expression.** Uncropped blots corresponding to Fig.3 in the main article text. Boxed regions are used in the main figures.

1. Collagen with the corresponding β-actin for Fig. 3 A


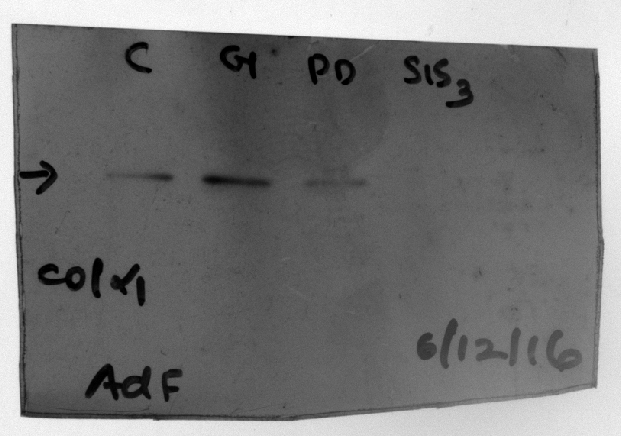

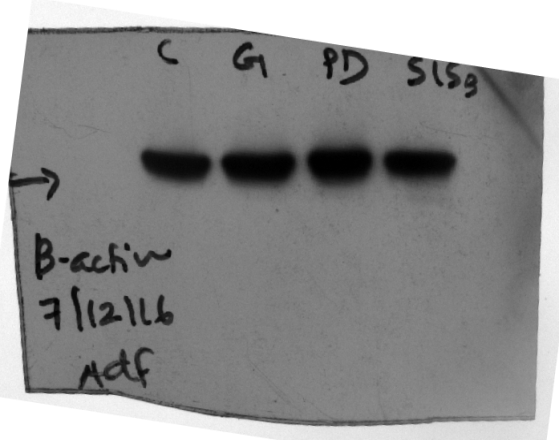


1. DDR2 with the corresponding β-actin for Fig. 3 C


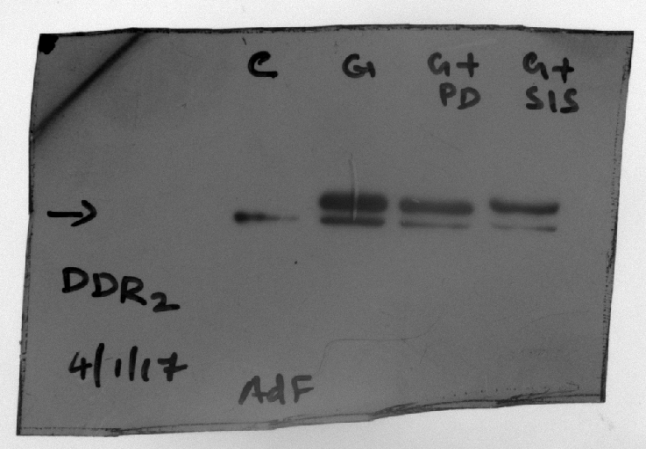

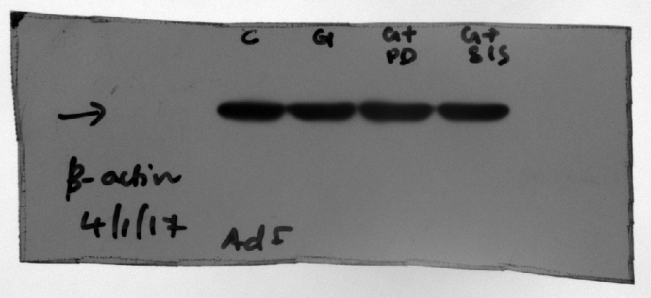


**ERK1/2 MAPK mediates DDR2-dependent collagen gene expression in HG-treated cells.**Uncropped blots corresponding to Fig.4 in the main article text. Boxed regions are used in the main figures.

1. Collagen with the corresponding β-actin for Fig. 4 A


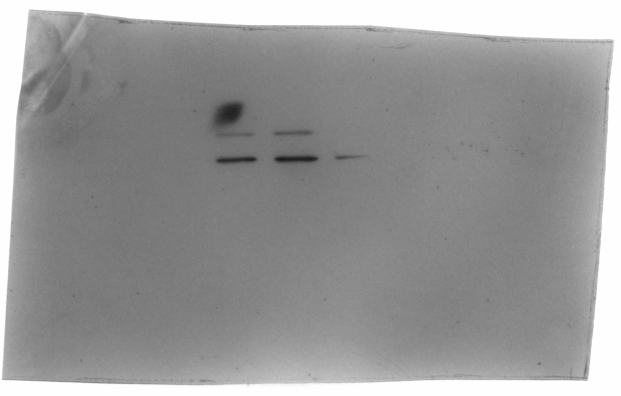

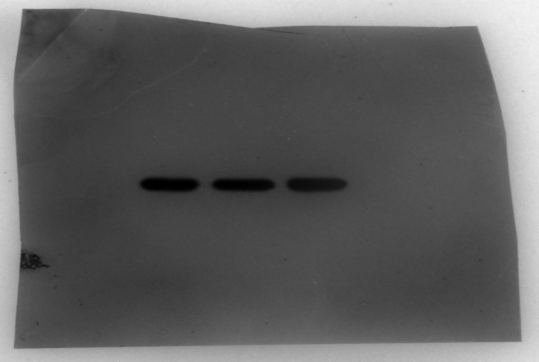


Collagenα1 type 1

1. DDR2 with the corresponding β-actin for Fig. 4 C


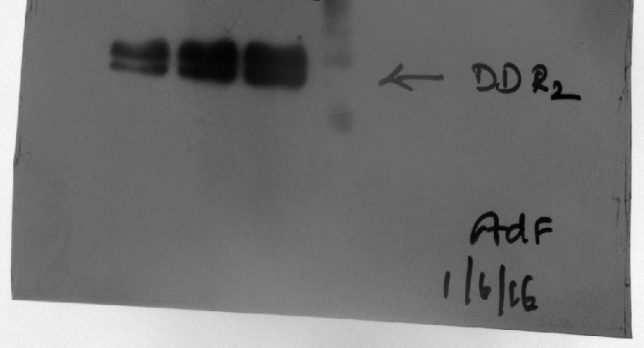

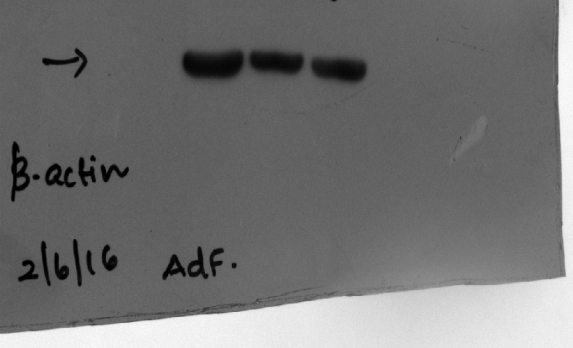


1. PhosphoERK1/2 withthecorresponding total ERK1/2 and β-actin for Fig. 4 E


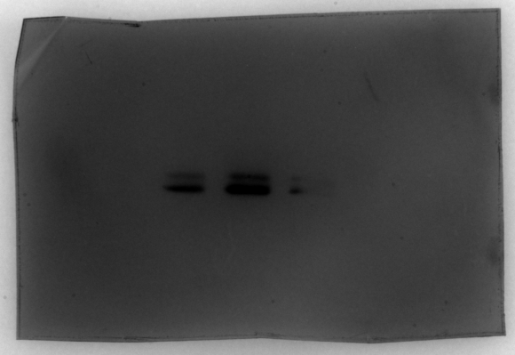

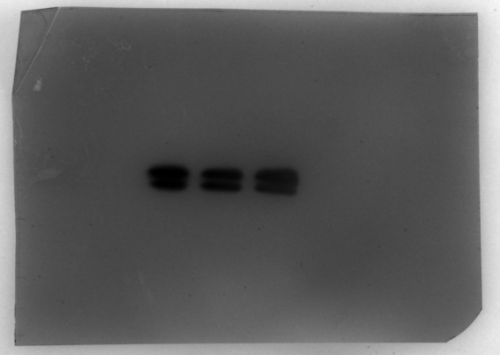


Total ERK1/2

p- ERK1/2


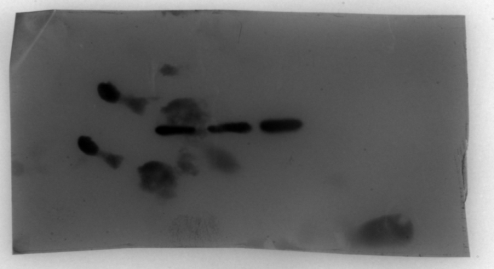


β-actin

**Resveratrol prevents HG-induced collagen type I and DDR2 in vascular adventitial fibroblasts.**Uncropped blots corresponding to Fig.5 in the main article text. Boxed regions are used in the main figure.

1. Collagen with the corresponding β-actin for Fig. 5 A


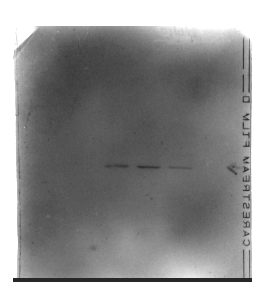

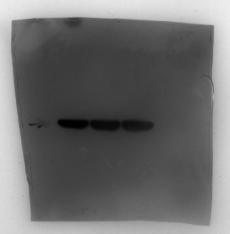


1. DDR2 with the corresponding β-actin for Fig. 5 C


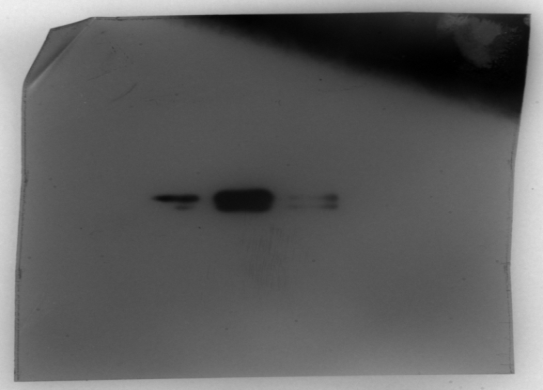

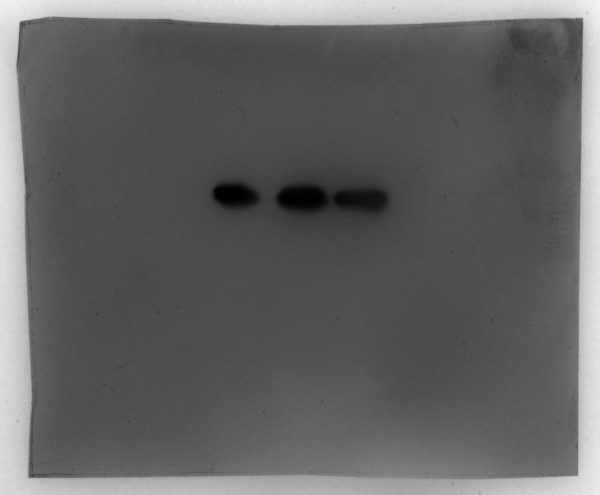


1. TGF-β with the corresponding β-actin for Fig. 5 E


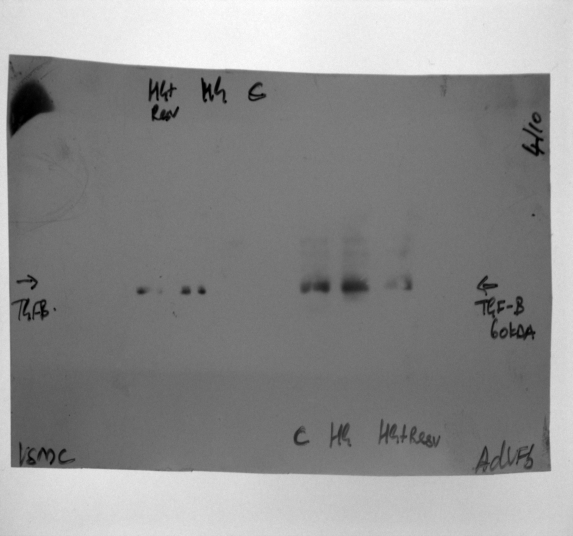

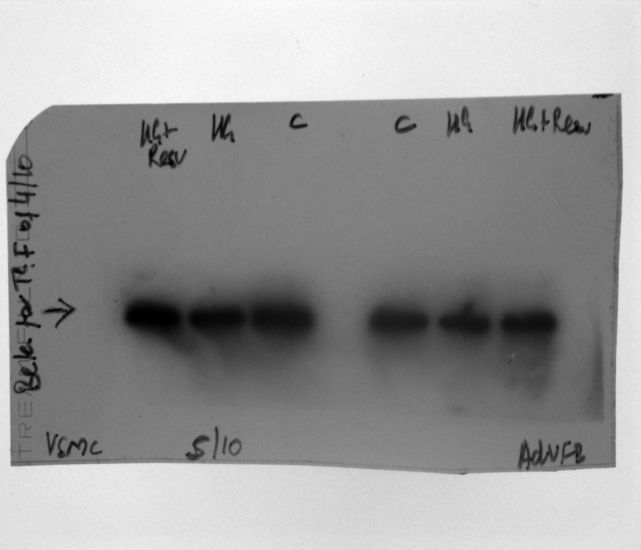


1. Collagen, DDR2 and the corresponding β-actin for Fig. 5 G


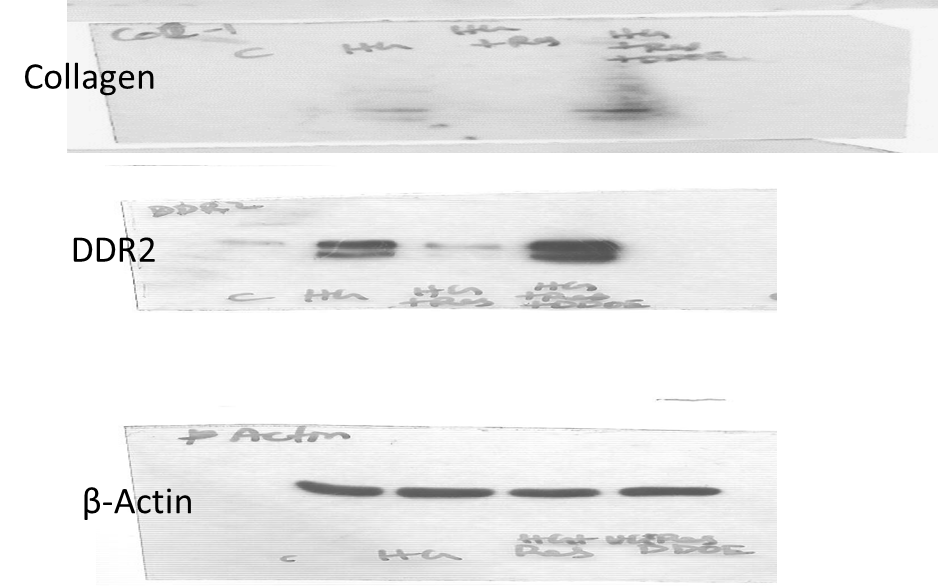


1. Collagen, DDR2 and the corresponding β-actin for Fig. 5 I


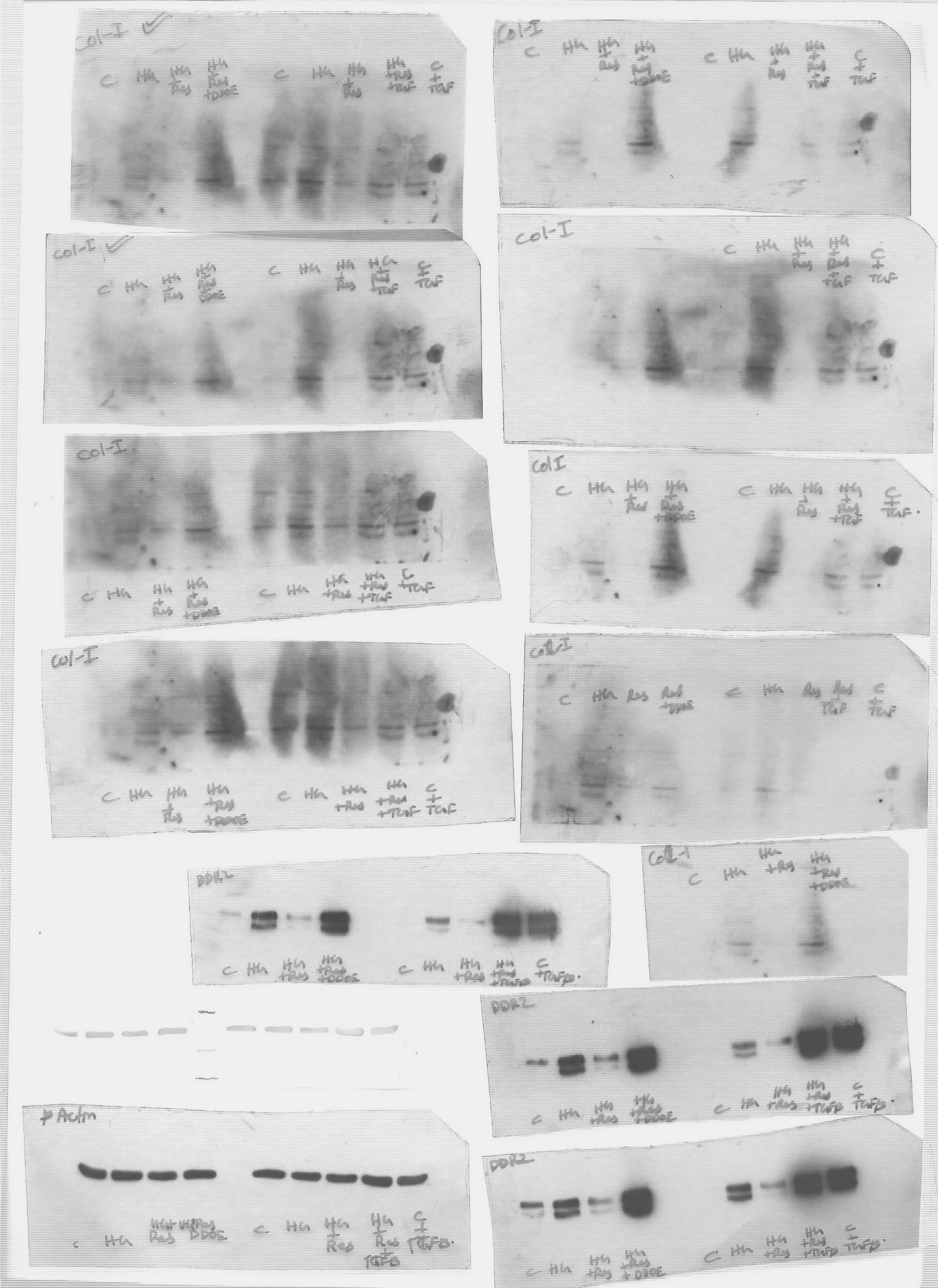

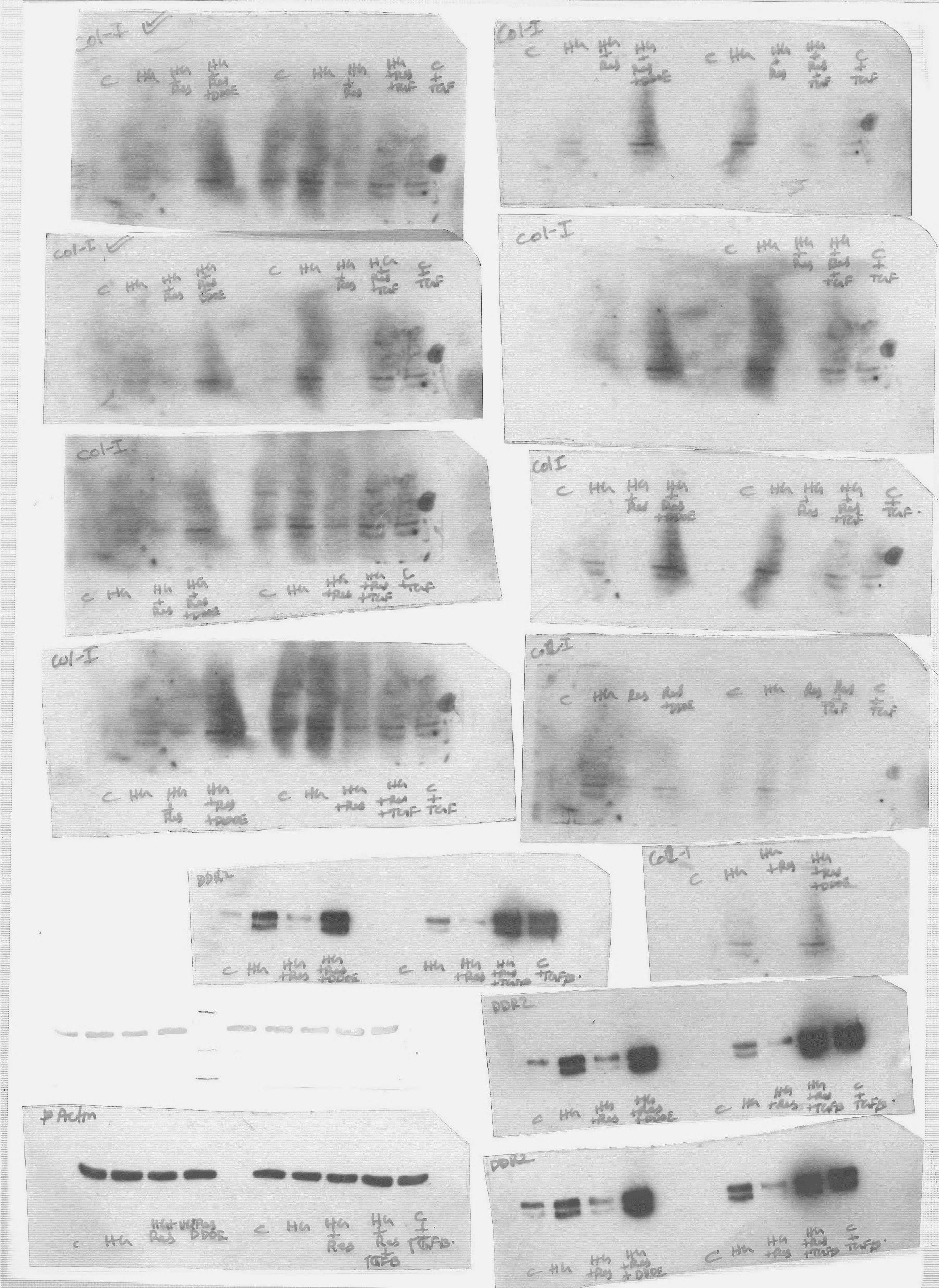


DDR2

β-Actin


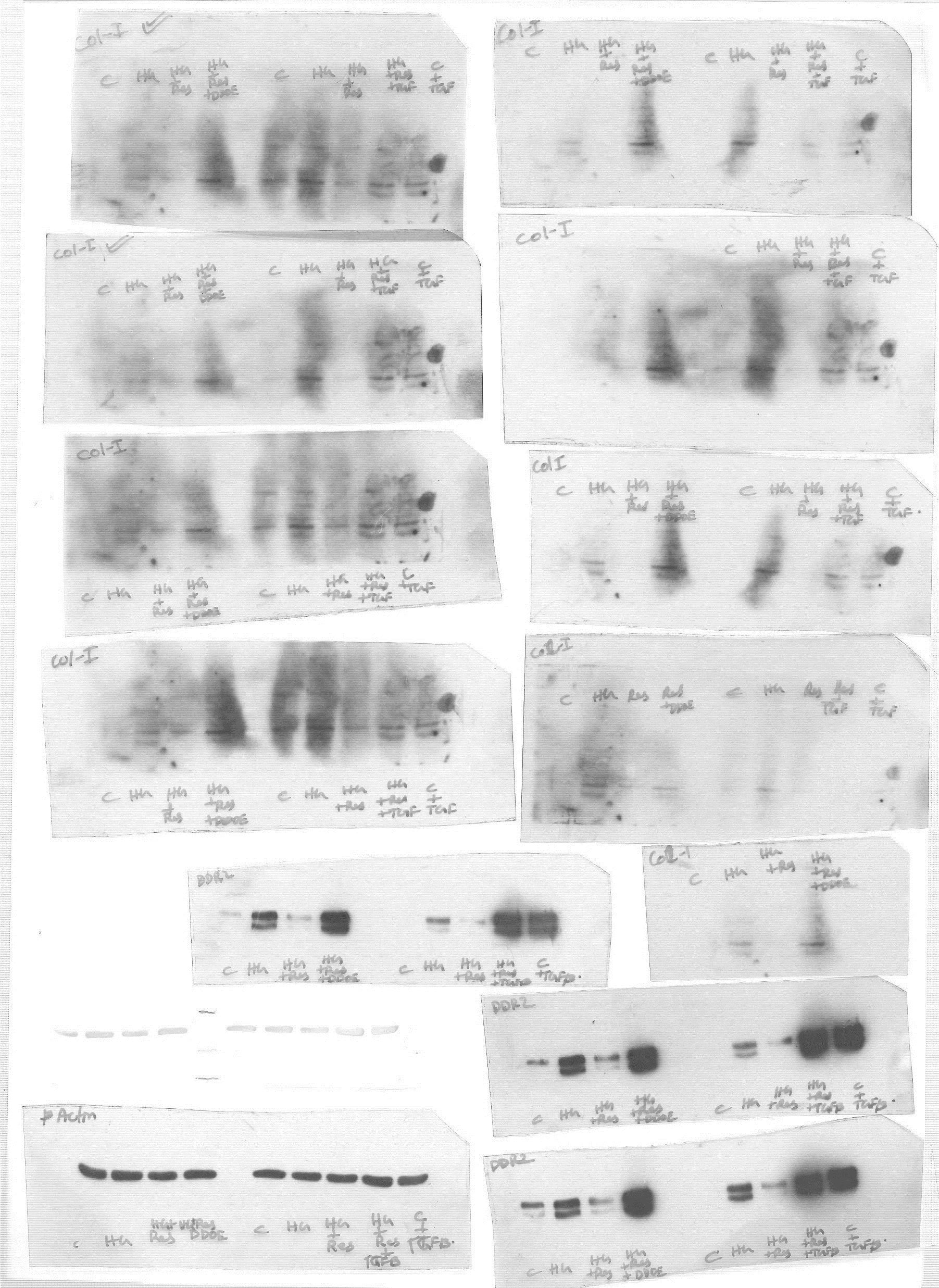


Collagen

**HG stimulates collagen type I and DDR2 in vascular smooth muscle cells.**Uncropped blots corresponding to Fig.6 in the main article text. Boxed regions are used in the main figures.

1. Collagen with the corresponding β-actin for Fig. 6 C


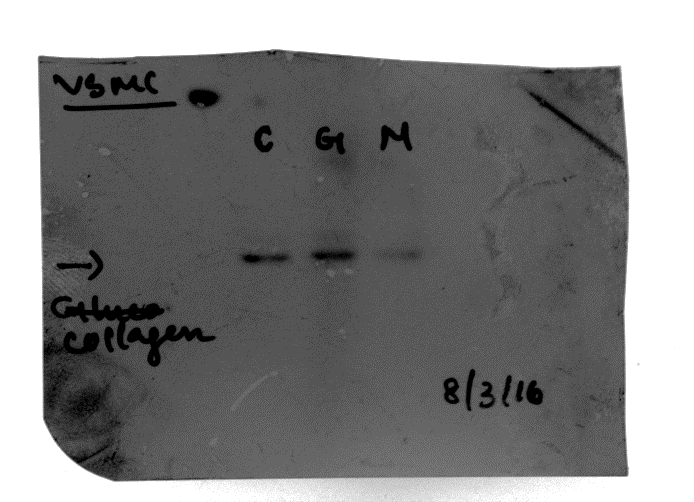

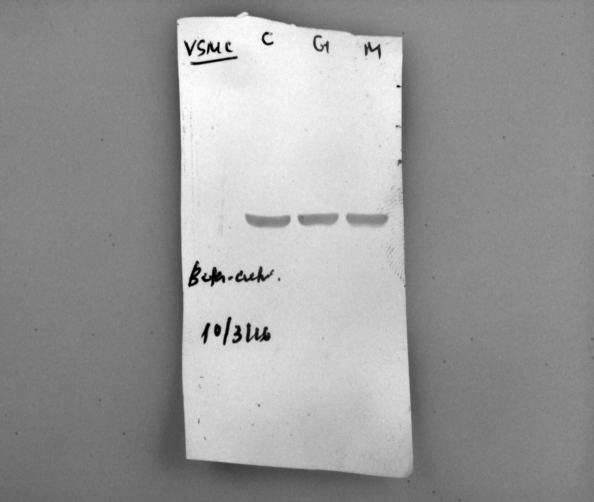


1. DDR2 with corresponding β-actin for Fig. 6 E


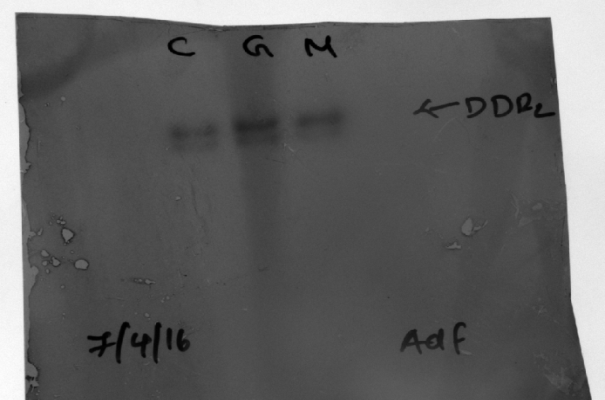

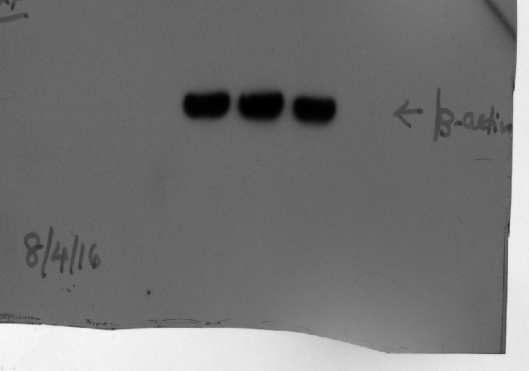


1. DDR2 siRNA validation and Collagen protein expression with the corresponding β-actin for Fig. 6 G


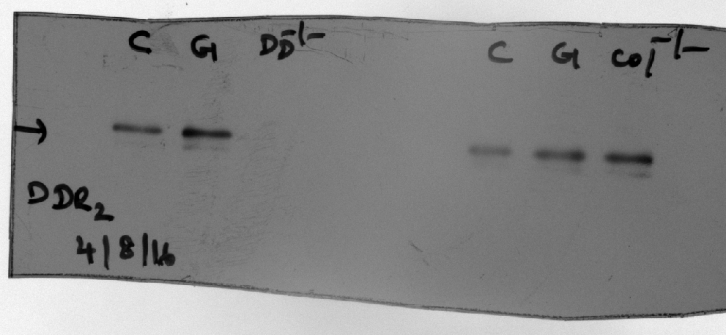


DDR2 siRNA validation


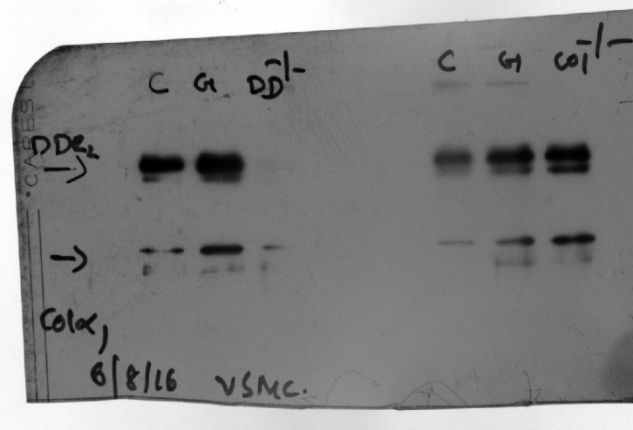


Collagen α1 type 1

**(Note: The DDR2 bands observed above collagen bands appear from the previous exposure of the membrane for DDR2 siRNA validation)**


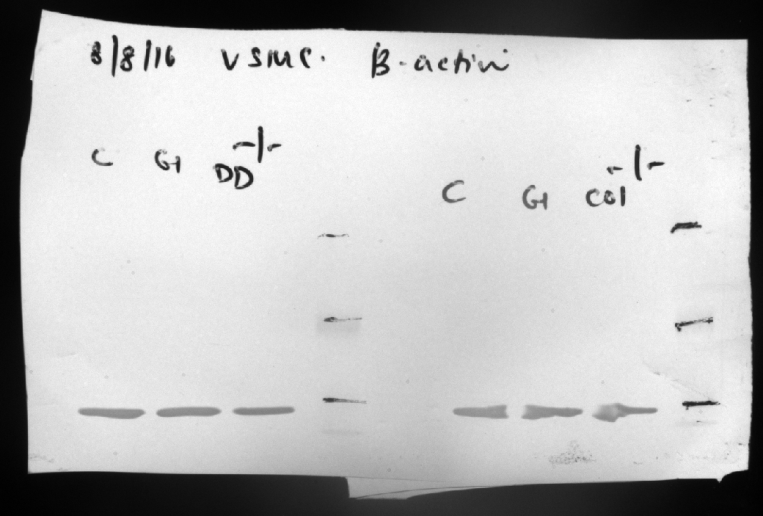


β-actin

**Figure 7: Resveratrol prevents HG-induced collagen type 1 and DDR2 in vascular smooth muscle cells.** Uncropped blots corresponding to Fig.7 in the main article text. Boxed regions are used in the main figures.

1. Collagen with the corresponding β-actin for Fig. 7A


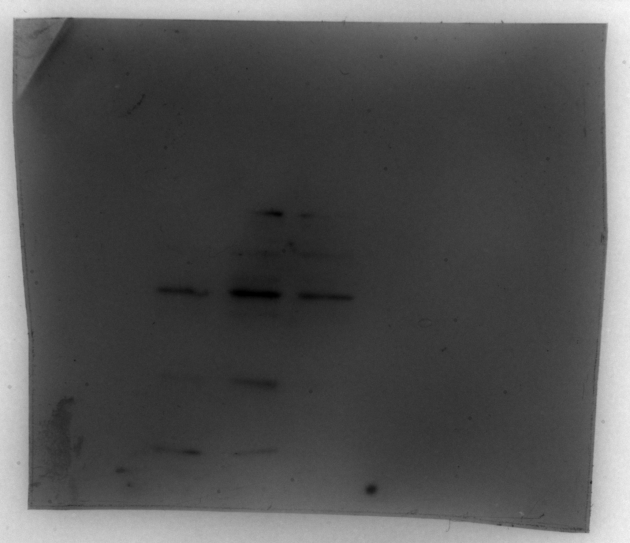

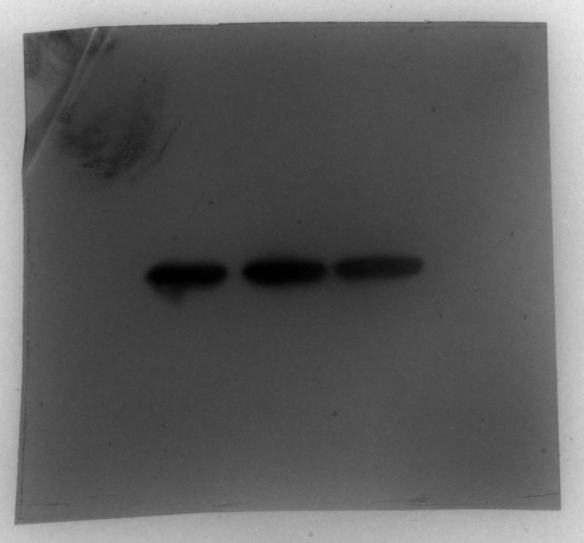


Collagenα1 type 1

1. DDR2 with the corresponding β-actin for Fig. 7C


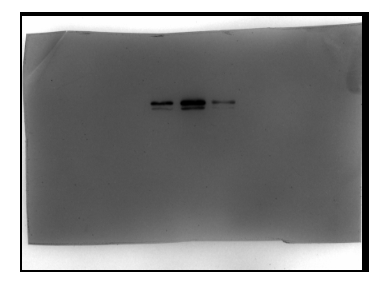

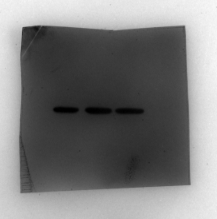


1. Collagen, DDR2 and corresponding β-actin for Fig. 7E

**
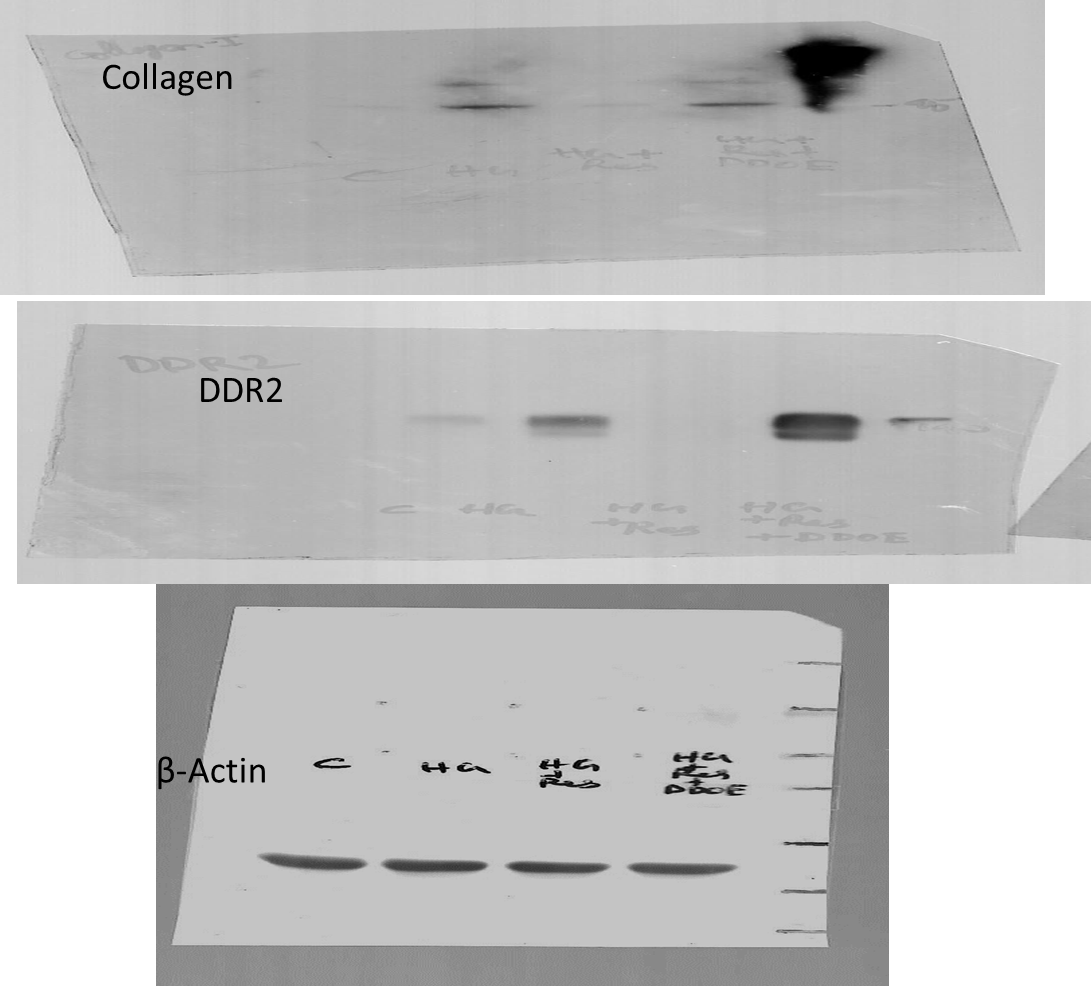
**

1. TGF-β with the corresponding β-actin for Fig. 7G

**
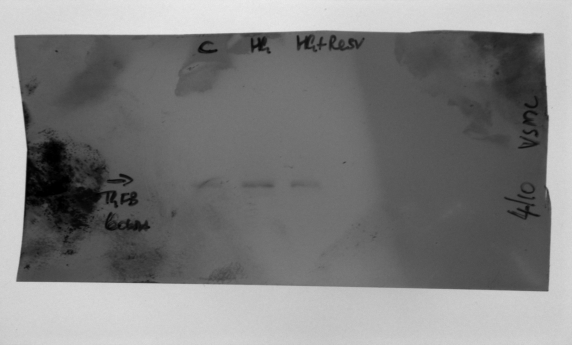

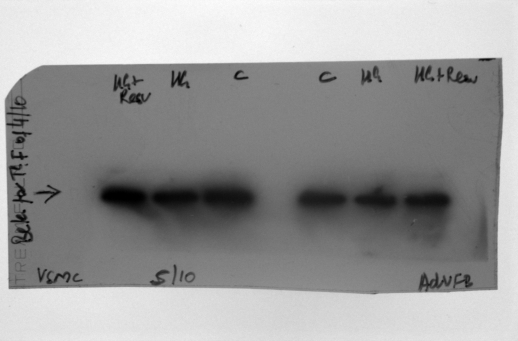
**

1. Collagen , DDR2 with corresponding β-actin for Fig.7I

**
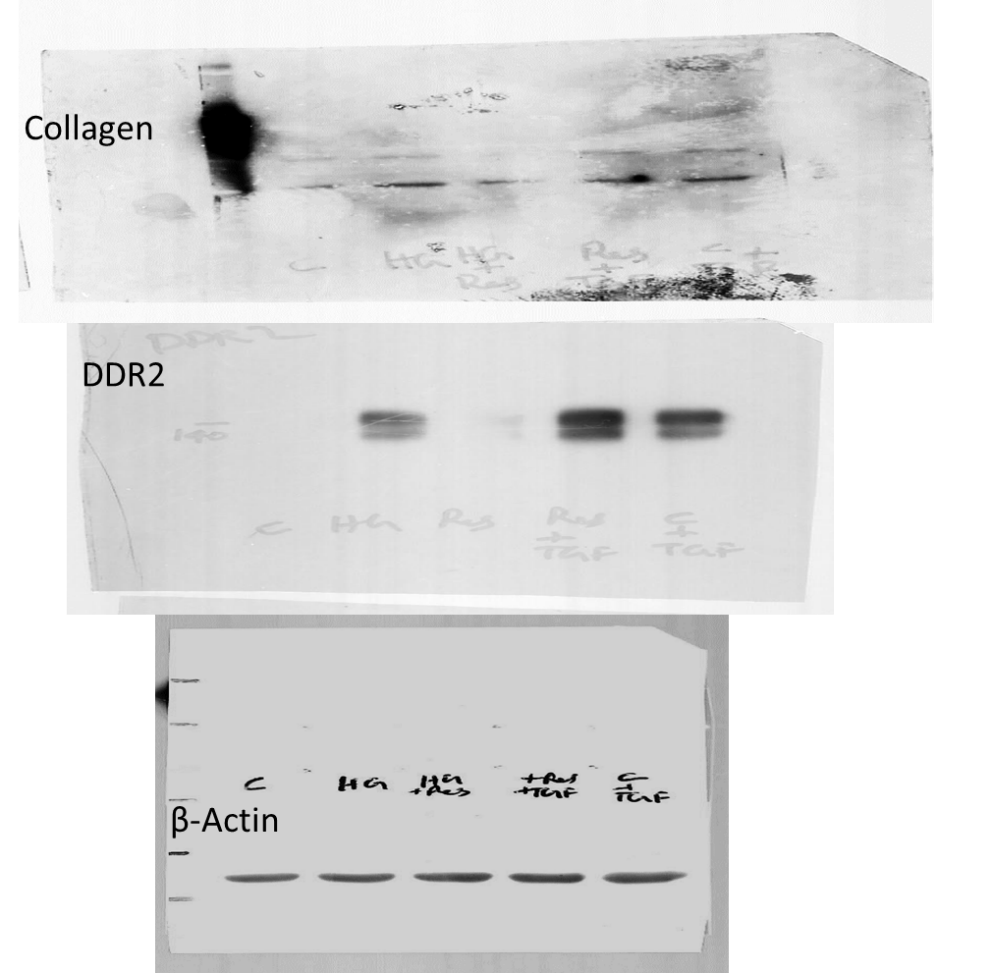
**

**Fig 9 : Western blot analysis of DDR2, Collagen types I and III in the abdominal aortic wall.**

DDR2 with the corresponding beta-actin for Fig 9M

**
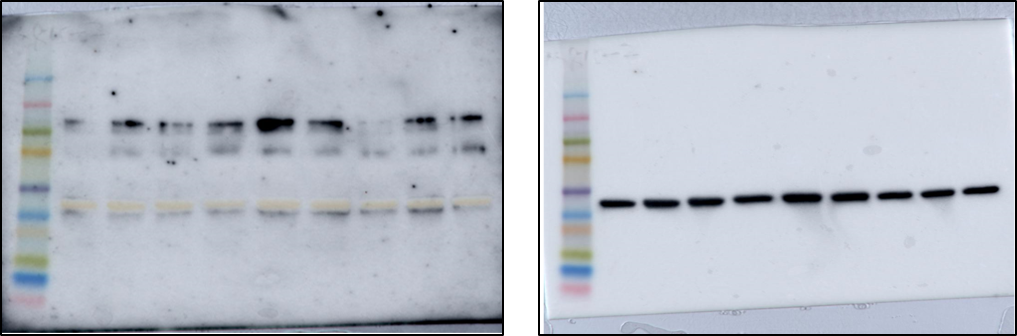
**

Collagen type I with the corresponding beta-actin for Fig 9K

**
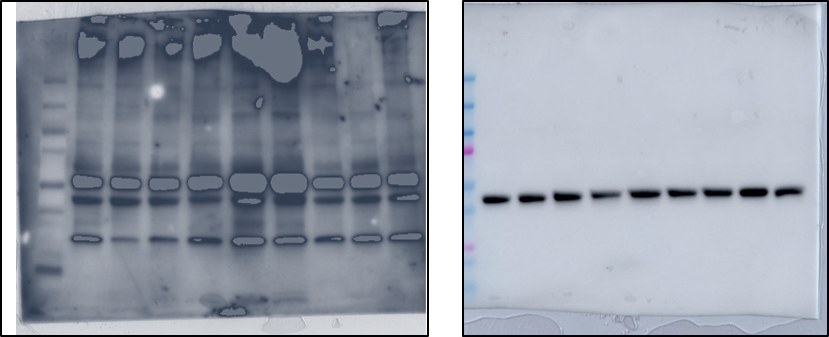
**

Collagen type III with the corresponding beta-actin for Fig 9I


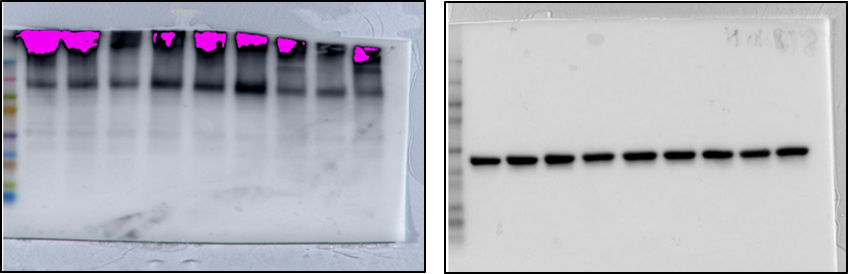


**Full-length blots corresponding to supplementary Fig. S1**

Boxed regions were used in the representative figures.

**1.**Collagen blot with the corresponding β-actin for **Fig. S1 A**

**
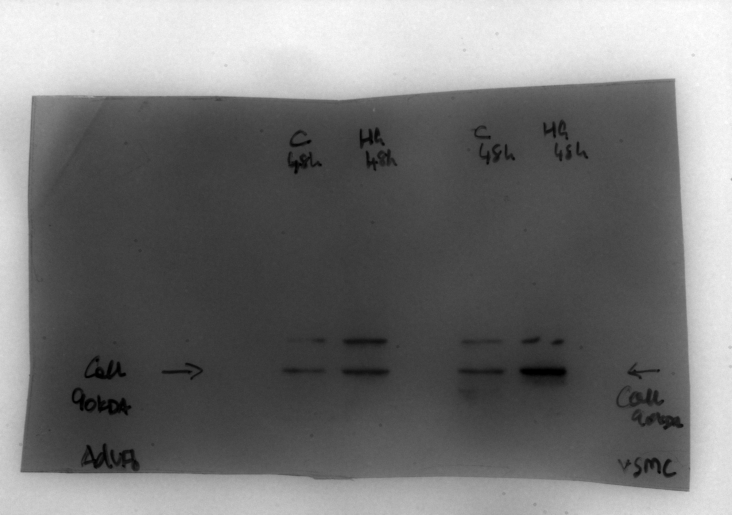

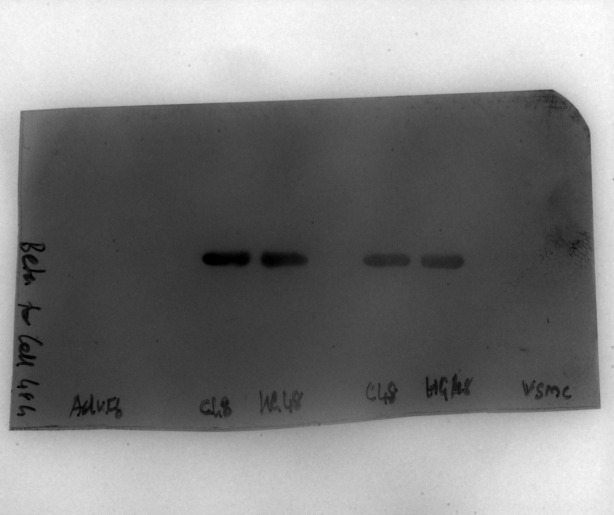
**

**2.**Collagen blot with the corresponding β-actin for **Fig. S1 C**

**
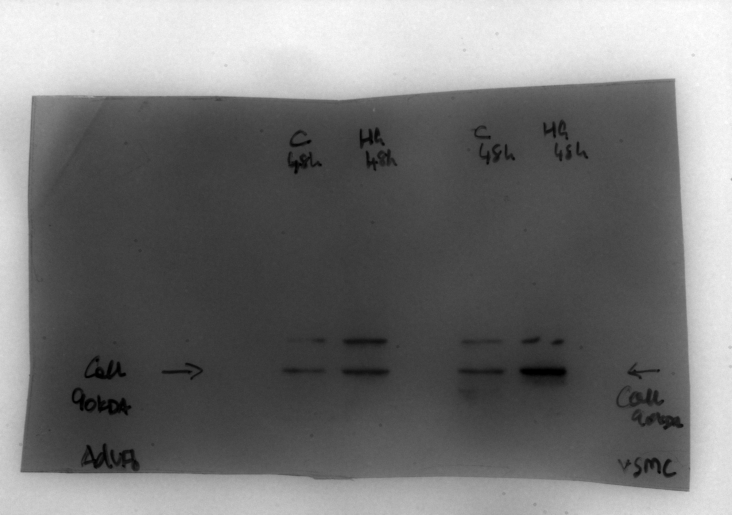

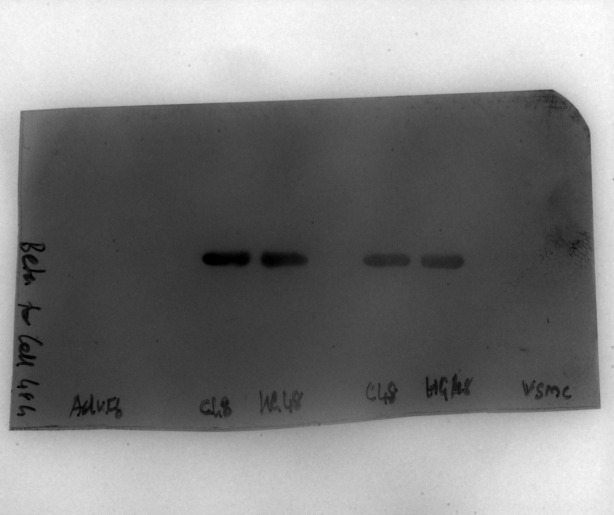
**

**3.**DDR2 blot with the corresponding β-actin for Fig. **S1 B.**The blots were rotated horizontally for representation.


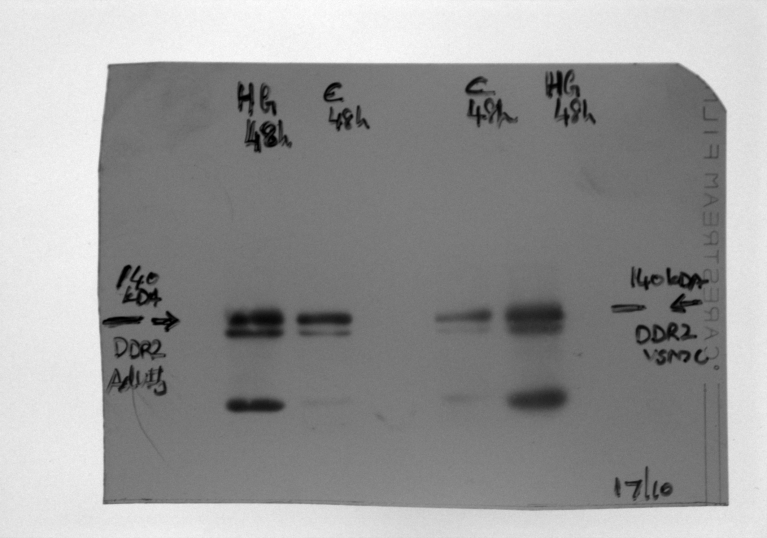

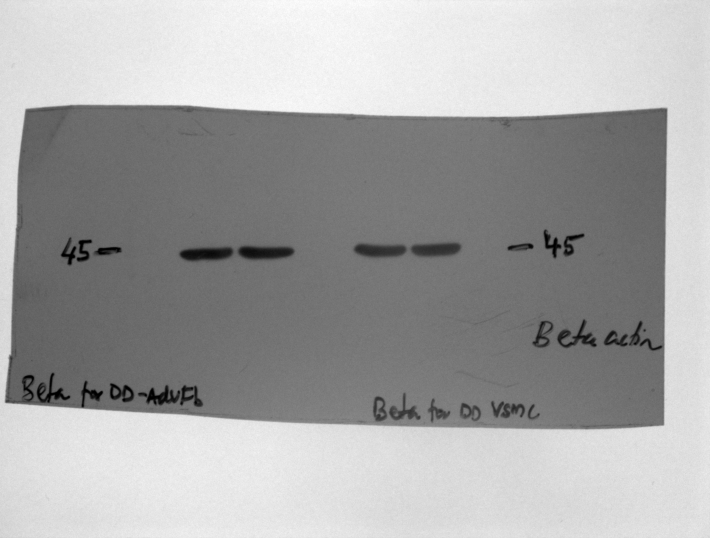


4. DDR2 blot with the corresponding β-actin for **Fig. S1 D**


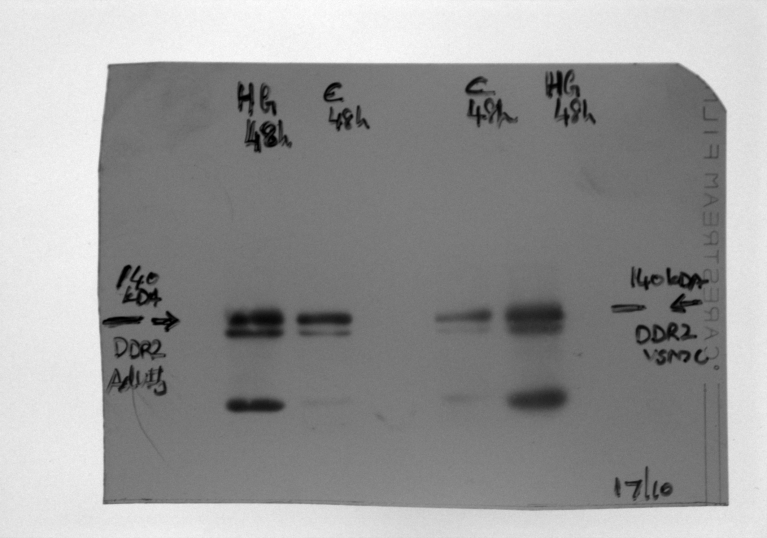

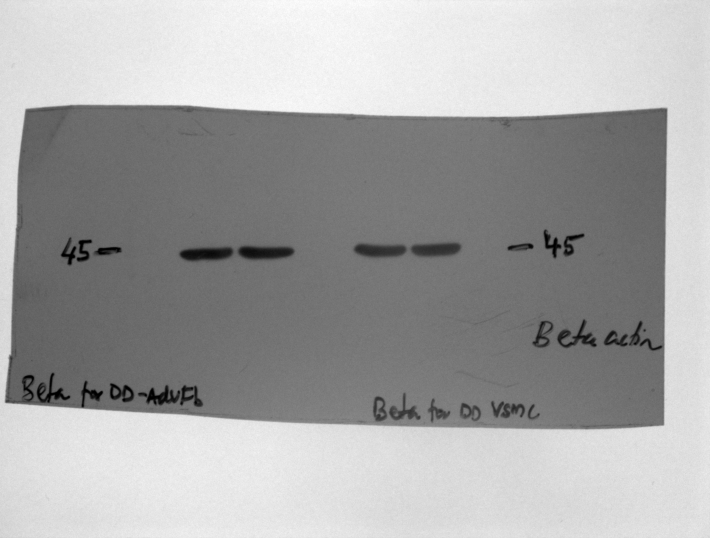


5. DDR2, collagen and their corresponding β-actin blots for **Fig. S1 E**


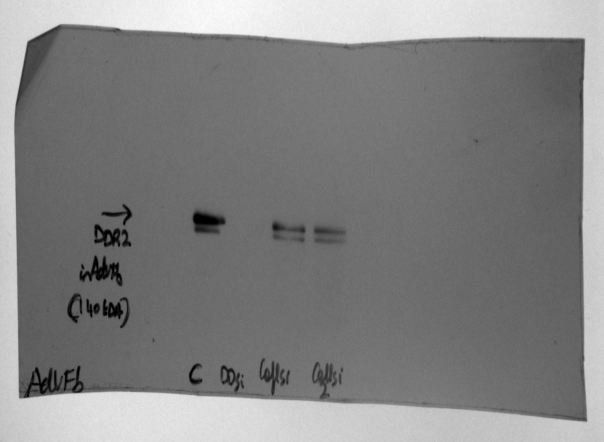

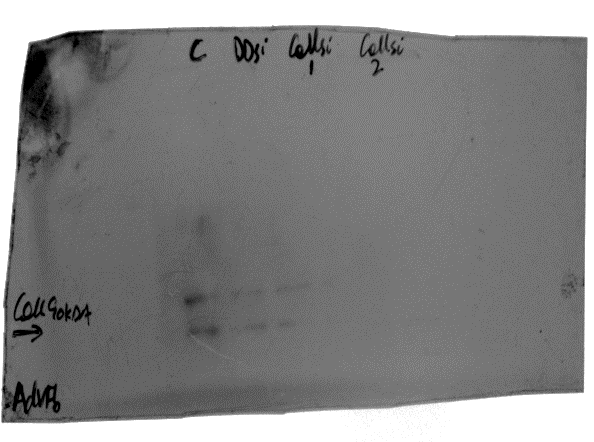


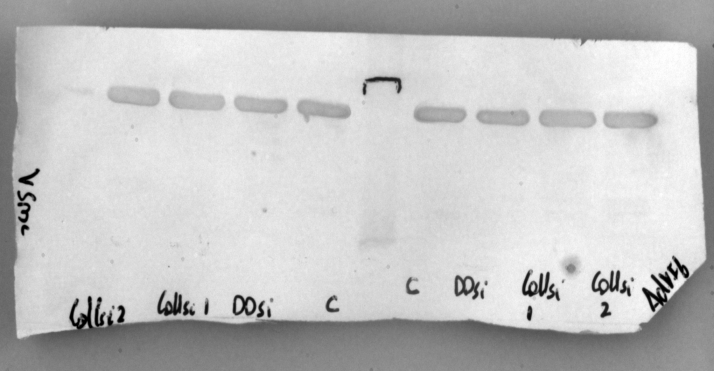


**6.** DDR2, collagen and their corresponding β-actin blots for **Fig. S1 F**


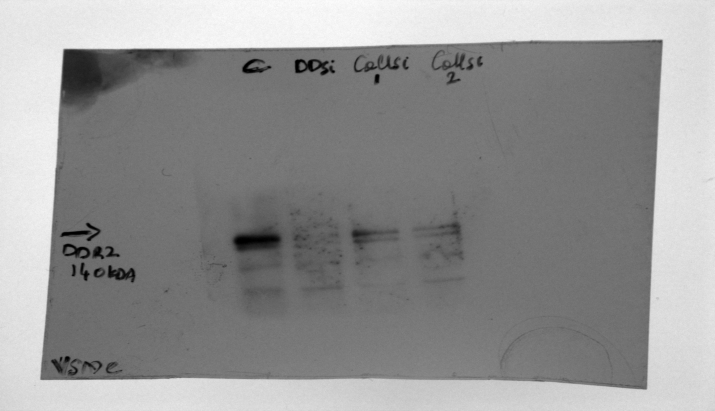

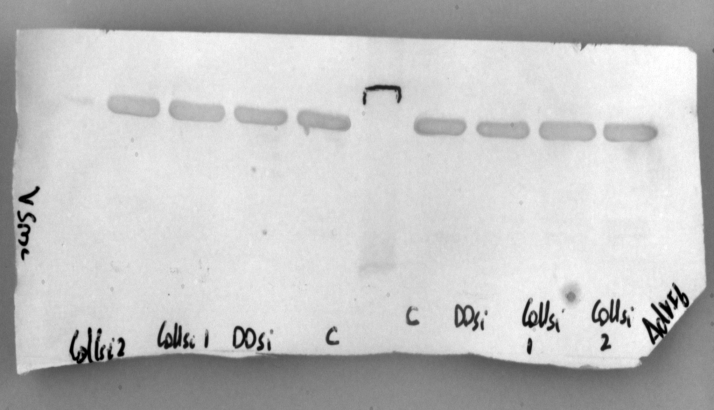

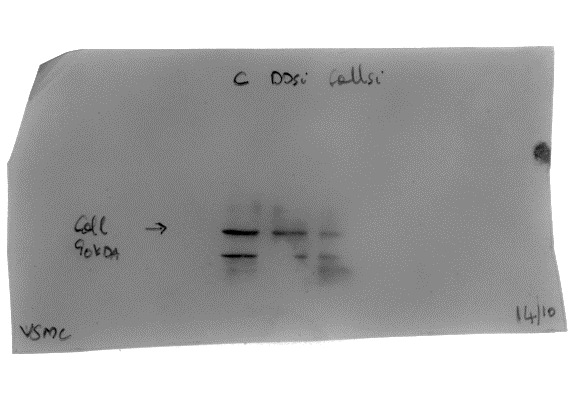

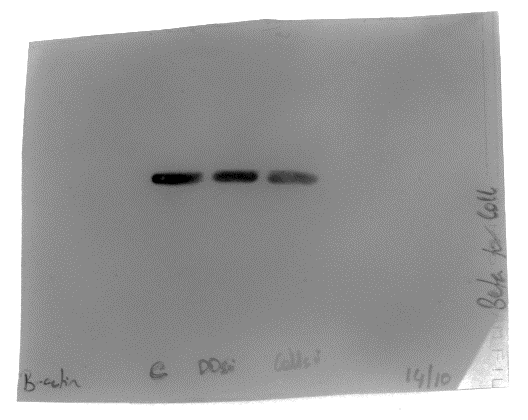


7. TGF-β1, DDR2, collagen and their corresponding β-actin blots for **Fig S1 G.** The collagen and its corresponding β-actin blot were rotated horizontally for representation.

**
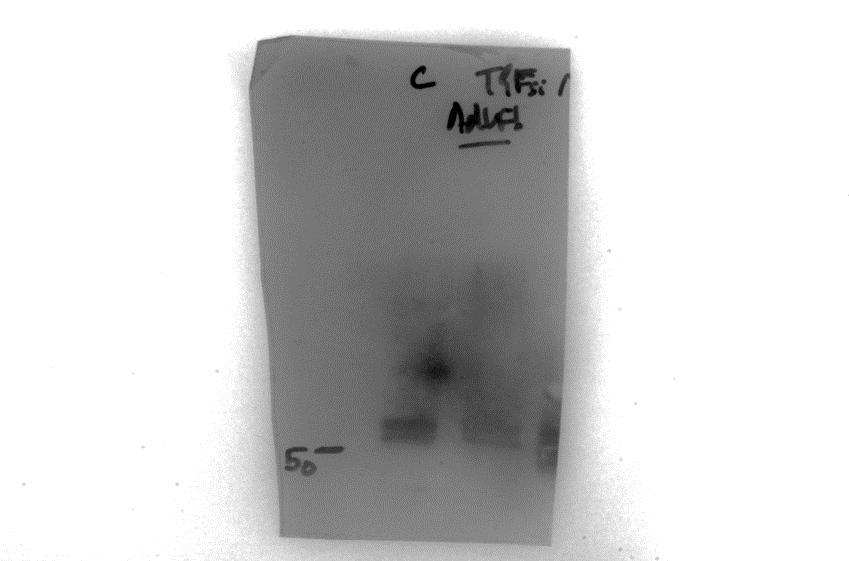

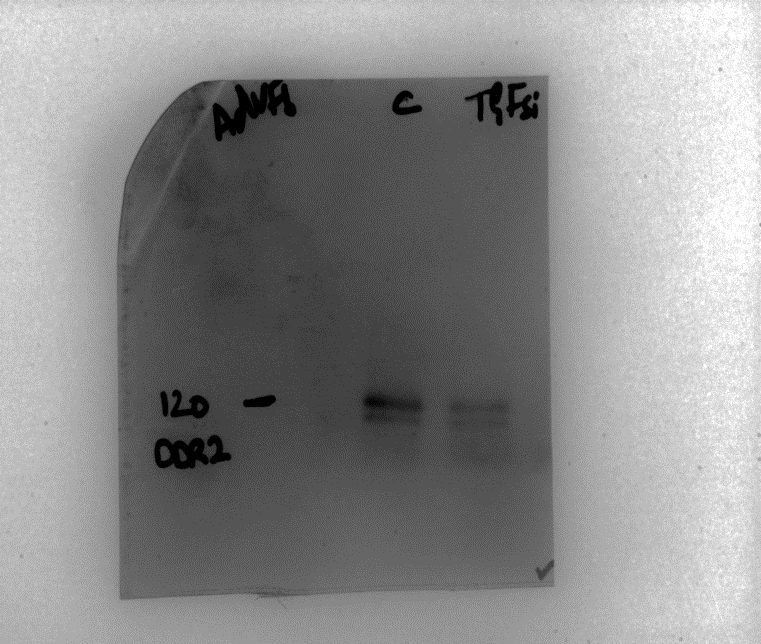

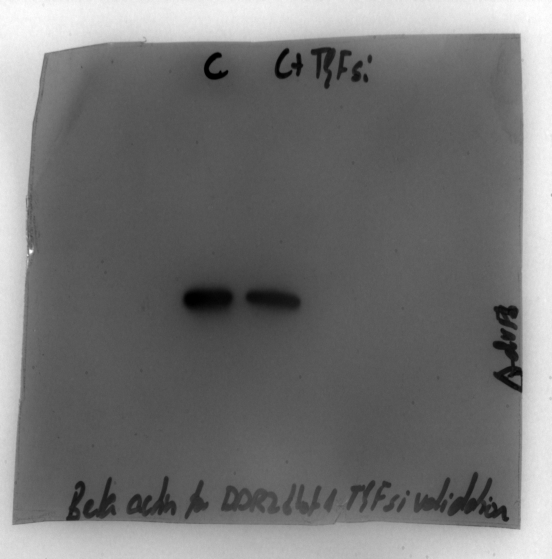
**


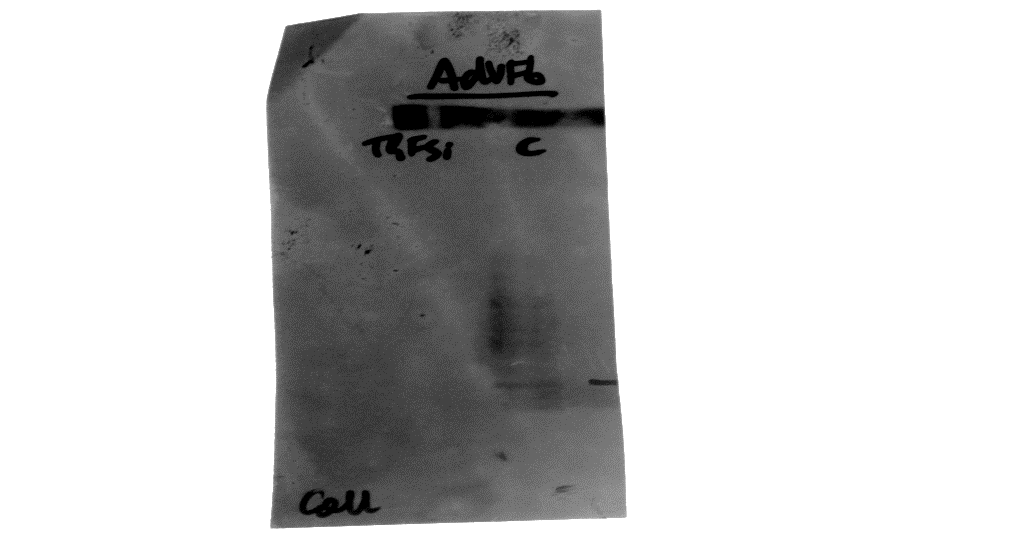

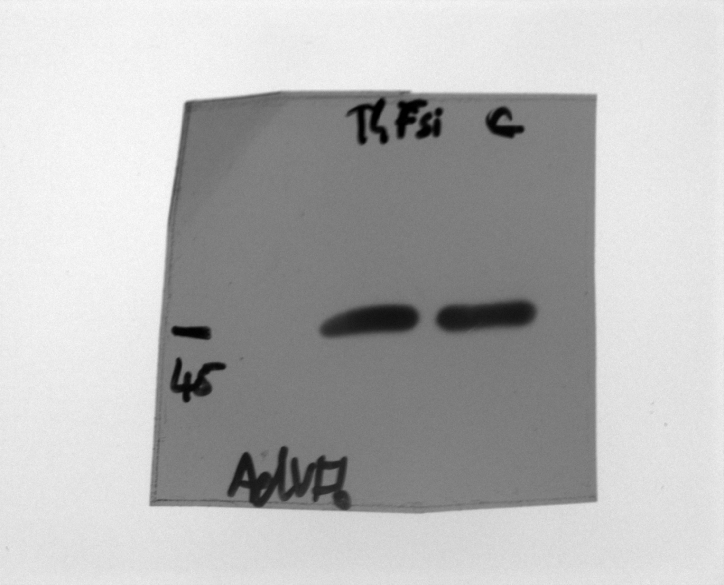


8**.**Collagen with the corresponding β-actin blot for **Fig. S1 H**


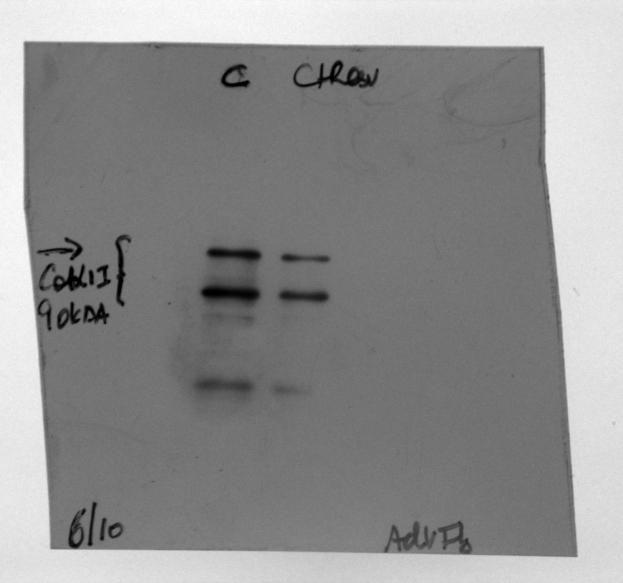

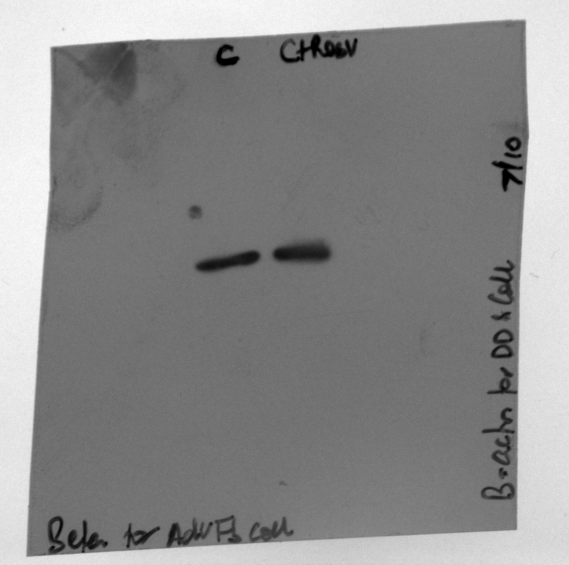


10.DDR2 with the corresponding β-actin for **Fig. S1 I**


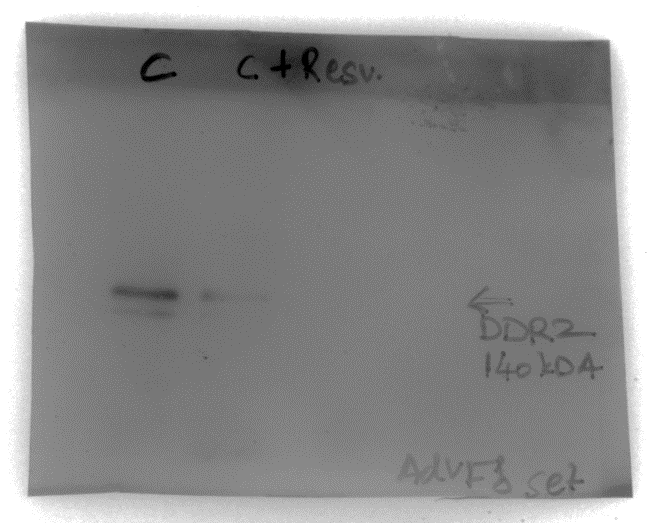

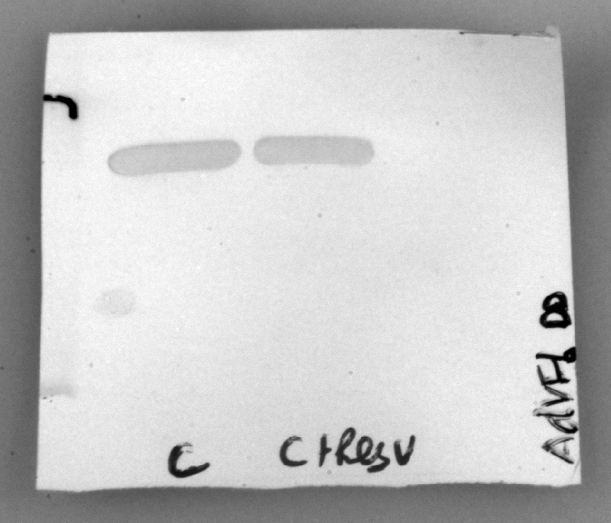


12. TGF-β1 with the corresponding β-actin for **Fig. S1 J.**


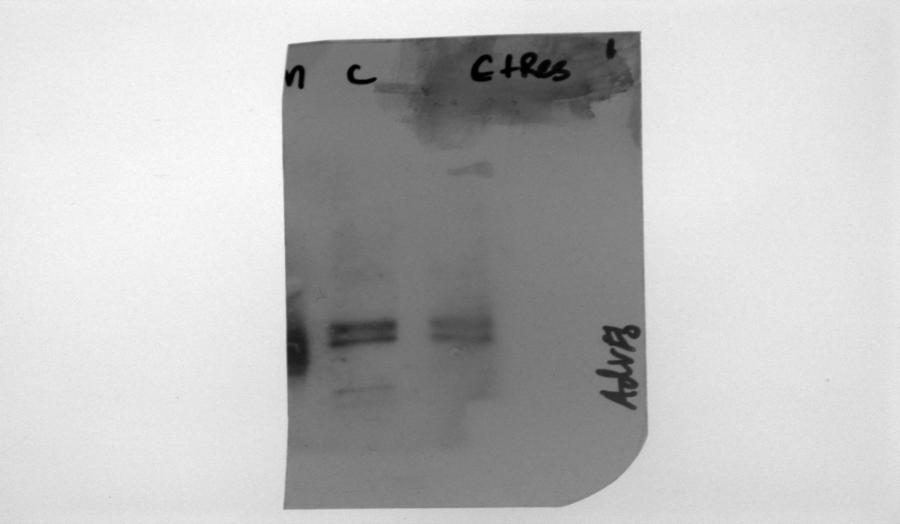

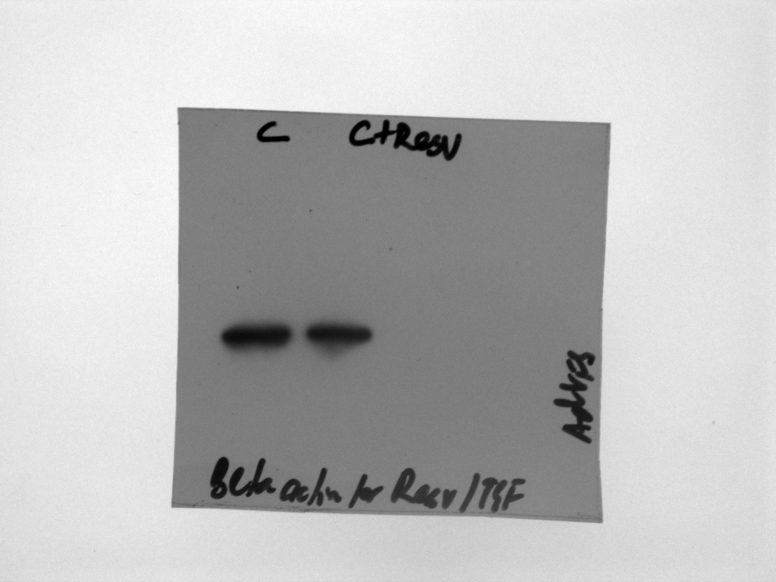


9**.**Collagen with the corresponding β-actin for **Fig. S1 K**. The β-actin blot was rotated horizontally for representation.


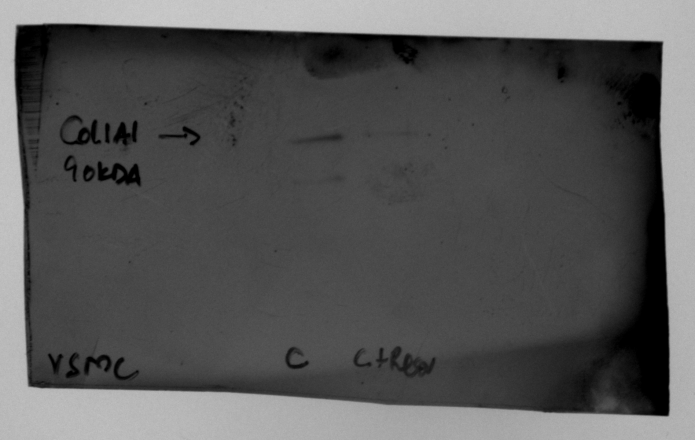

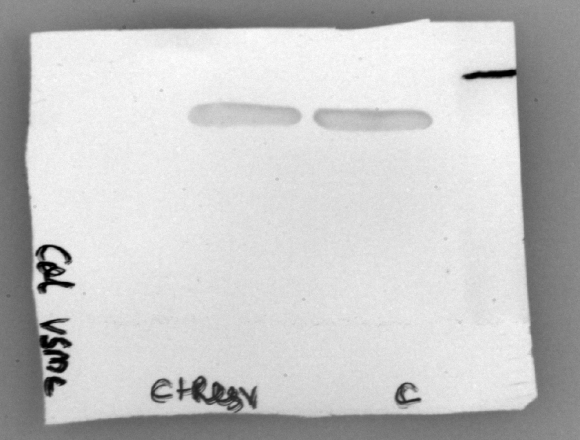


11. DDR2 with the corresponding β-actin for **Fig. S1 L**. The β-actin blot was rotated horizontally.
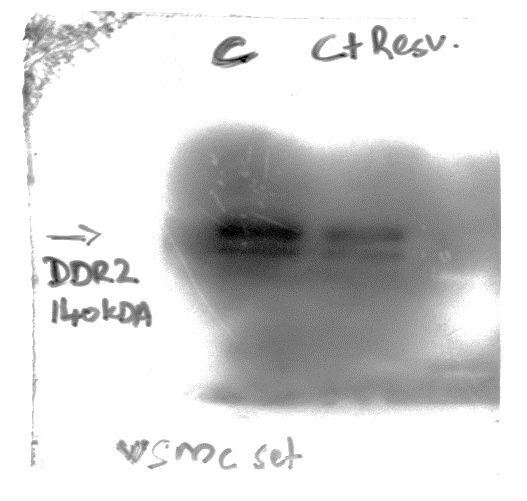

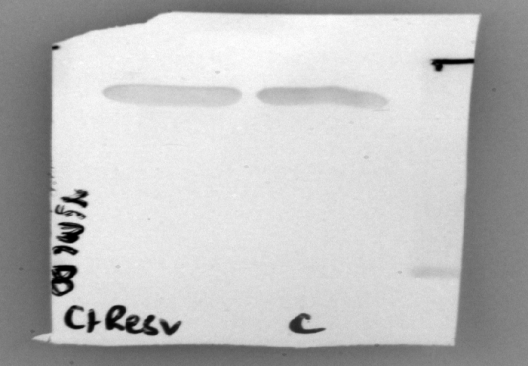

Supplement: S5 Fig — (DOCX) [file pone.0225911.s005.docx]
